# Multiple Growth Factor Targeting by Engineered Insulin-like Growth Factor Binding Protein-3 Augments EGF Receptor Tyrosine Kinase Inhibitor Efficacy

**DOI:** 10.1038/s41598-020-59466-6

**Published:** 2020-02-17

**Authors:** Elizabeth A. Wang, Wan-Yu Chen, Chi-Huey Wong

**Affiliations:** 1 Genomics Research Center, Academia Sinica, Taipei, 11529 Taiwan; 2 Department of Chemistry, The Scripps Research Institute, La Jolla, CA 92037 USA

**Keywords:** Biochemistry, Cytokines, Cancer, Cancer therapy

## Abstract

Resistance to cancer therapy is a challenge because of innate tumor heterogeneity and constant tumor evolution. Since the pathway of resistance cannot be predicted, combination therapies may address this progression. We discovered that in addition to IGF1 and IGF2, IGFBP-3 binds bFGF, HGF, neuregulin, and PDGF AB with nanomolar affinity. Because growth factors drive resistance, simultaneous inhibition of multiple growth factor pathways may improve the efficacy of precision therapy. Growth factor sequestration by IGFBP-3-Fc enhances the activity of EGFR inhibitors by decreasing cell survival and inhibiting bFGF, HGF, and IGF1 growth factor rescue and also potentiates the activity of other cancer drugs. Inhibition of tumor growth *in vivo* with adjuvant IGFBP-3-Fc with erlotinib versus erlotinib after treatment cessation supports that the combination reduces cell survival. Inhibition of multiple growth factor pathways may postpone resistance and extend progression-free survival in many cancer indications.

## Introduction

The understanding of oncogenic driver mutations has led to the development of many successful targeted therapies in cancer. However, resistance to the original drug inevitably develops during treatment. Resistance stems from changes in multiple pathways^1–3^, one of which is increased growth factor signaling. The growth factors fibroblast growth factor (FGF), epidermal growth factor (EGF), hepatocyte growth factor (HGF), insulin-like growth factor (IGF), and neuregulin-1 (NRG) rescue oncogene addicted cancer cell lines from various TKIs, and growth factor rescue contributes to both intrinsic and acquired resistance^2–4^. Inhibition of multiple pathways will more effectively combat the numerous mechanisms of resistance and tumor heterogeneity^5^.

The IGF system is important in cell growth and in carcinogenesis and tumor progression of different cancer cell types^6,7^. Availability of insulin-like growth factor-1 (IGF1) and insulin-like growth factor-2 (IGF2) is regulated by six related insulin-like growth factor binding proteins (IGFBP)^8^. IGFBP-3 binds IGF1 and IGF2, and IGF sequestration inhibits cellular proliferation; IGF release by proteolytic cleavage of IGFBP-3 can stimulate proliferation^9,10^. IGFBP-3 also exhibits an IGF-independent inhibition of proliferation and has multiple binding partners, some of which affect proteolysis^11–13^. A comprehensive review^11^ demonstrates the lack of consensus between IGFBP-3 expression levels and cancer incidence, disease severity, or prognostic survival, although reduced IGFBP-3 expression has been correlated with drug resistance^14–16^, and IGFBP-3 dosed twice daily reduced tumor growth *in vivo*^17,18^.

For the first time, we demonstrate that IGFBP-3 binds directly to multiple growth factors - bFGF, PDGF, HGF, VEGF-B, and NRG - in the nanomolar range by surface plasmon resonance and correspondingly inhibits ligand-stimulated proliferation. bFGF, EGF, HGF, IGF1, NRG, and PDGF are widely expressed in tumors^4^, so the discovery of a single protein that inhibits multiple growth factors has enormous potential in cancer treatment. To enhance its stability and therapeutic potential, we deleted protease sensitive sites from IGFBP-3 and constructed Fc fusions. We also added a high affinity VEGF domain (VEGF-trap) to an IGFBP-3 Fc construct to inhibit vascularization. Lastly, we show that an IGFBP-3 Fc construct in combination with EGFR targeted therapy augments inhibition of cancer cells both *in vitro* and *in vivo*. This construct with multiple growth factor inhibition properties, combined with targeted therapy, may improve efficacy and postpone the development of resistance in many existing and future cancer treatments.

## Results

### IGFBPs bind growth factors in addition to IGF1 and IGF2

Surface plasmon resonance was used to analyze binding of various growth factors to commercially available IGFBP-2, 3, and 7 immobilized to a CM4 surface. Binding to IGF1 and IGF2 is consistent with published values^19,20^. IGFBP-3 binds NRG, HGF, PDGF AB, and VEGF-B with nanomolar affinity; binding is specific, as not all binding proteins show affinity for these growth factors (Table 1). In addition, IGFBP-2, IGFBP-3, and IGFBP-7 do not bind aFGF, CXCL-16, HB-EGF, IL-8, MIP1α, MIP1β, PlGF, VEGF-A 165, VEGF-C, and VEGF-D (data not shown). Sensorgrams of HGF, NRG, and PDGF AB binding are shown in Supplementary Fig. S1, and association and dissociation constants are found in Supplementary Table S1.

**Table 1:** Affinity Constants of IGFBPs and BP3-Fc’s.

	IGF1	IGF2	bFGF	HGF	NRG	PDGF^b^	VEGF-B	VEGF
IGFBP-2	0.83 (0.48)	4.27 (1.92)	not done	no binding	no binding	no binding (AB)	2.82	no binding
IGFBP-3	0.23 (0.09)	0.34 (0.17)	1.12(0.33); 1.32*	1.44 (0.89)	2.09 (0.25)	3.75 (1.06) (AB)	16.1 (18.1)	no binding
IGFBP-7	no binding	no binding	not done	Complex^a^	1600 (431)	4.2 (4.88)(AB)	9.88 (9.93)	no binding
56662	0.27 (0.26)	0.1 (0.22)	2.37 (1.94); 6.58 (8.88)*	2.36 (2.11); 4.74 (3.81)*	4.46 (6.25); 4.66 (6.23)*	0.52 (BB); 4.19 (4.88)* (BB)	14.32 (12.73)	no binding
D3	0.51 (0.25)	0.22 (0.004)	3.29 (1.28)*	2.63 (1.82)*	2.62 (1.76)*	5.29 (1.98)* (BB)	19.75 (15.46)*	no binding
Chimera A	0.57 (0.59)	0.21 (0.26)	1.71 (0.91)	1.35 (1.82)*	3.21 (2.4); 1.71 (1.50)*	4.38 * (BB); 2.44 (2.7)* (AB)	0.49	0.0052^c^ (0.0078)

K_D_ units = nM; standard deviation in parentheses; the absence of s.d. indicates a single measurement.

*Growth factors were immobilized instead of the binding protein to reduce background.

^a^Interactions did not fit well to a simple 1:1 binding model; the presence of incompletely processed as well as mature HGF (expressed in insect cells) would result in heterogeneous binding.

^b^PDGF AB and PDGF BB used as noted.

^c^k_d_ is very low and difficult to measure.

### Construction of Fc dimers

Expanding our study of IGFBP-3 because of its unique binding properties, we added the Fc portion of IgG1 to the C terminus of IGFBP-3 to increase serum half-life: this construct is 56662. Removal of protease sensitive sites in IGFBP-2 and IGFBP-4^21–23^ increased stability and enhanced inhibitory activity of IGFBP-2 and-4, so we deleted protease susceptible sites in the midregion of the IGFBP-3^24–29^ in 56662; two deletion constructs, h3t33 and D3, were chosen for further study after analysis for stability (see Supplementary Table S2), growth factor binding, and *in vitro* inhibitory activity. BP3-Fc will refer to unmodified or modified IGFBP-3 Fc constructs (56662, h3t33, or D3). Anti-angiogenic agents can prolong progression-free survival in several types of cancer, and we inserted VEGF-trap, a high affinity VEGF binding domain comprising of VEGF receptor 1 domain 2 and VEGF receptor 2 domain 3^30^, between the IGFBP-3 and Fc domain (chimera A) to further expand the anti-tumor activity. Complete amino acid sequences are listed in Supplementary Table S3.

### Binding analysis of Fc dimers

Surface plasmon resonance studies demonstrate that the BP3-Fc constructs show similar affinity to growth factors as IGFBP-3 (Table 1). Binding constants calculated with immobilized growth factor are noted with asterisks.

IGF1 binding is not affected by other ligands. Only IGF1 or 2 compete with biotinylated IGF1 in Elisa (Supplementary Fig. S1), and NRG binds in 50 nM IGF1 (Supplementary Fig. S1), both experiments suggesting non-overlapping binding domains. Saturating VEGF does not alter the affinity of chimera A for IGF-1 (0.4 nM alone versus 0.2 nM).

### BP3-Fc inhibits proliferation induced by multiple growth factors

**Figure 1:**
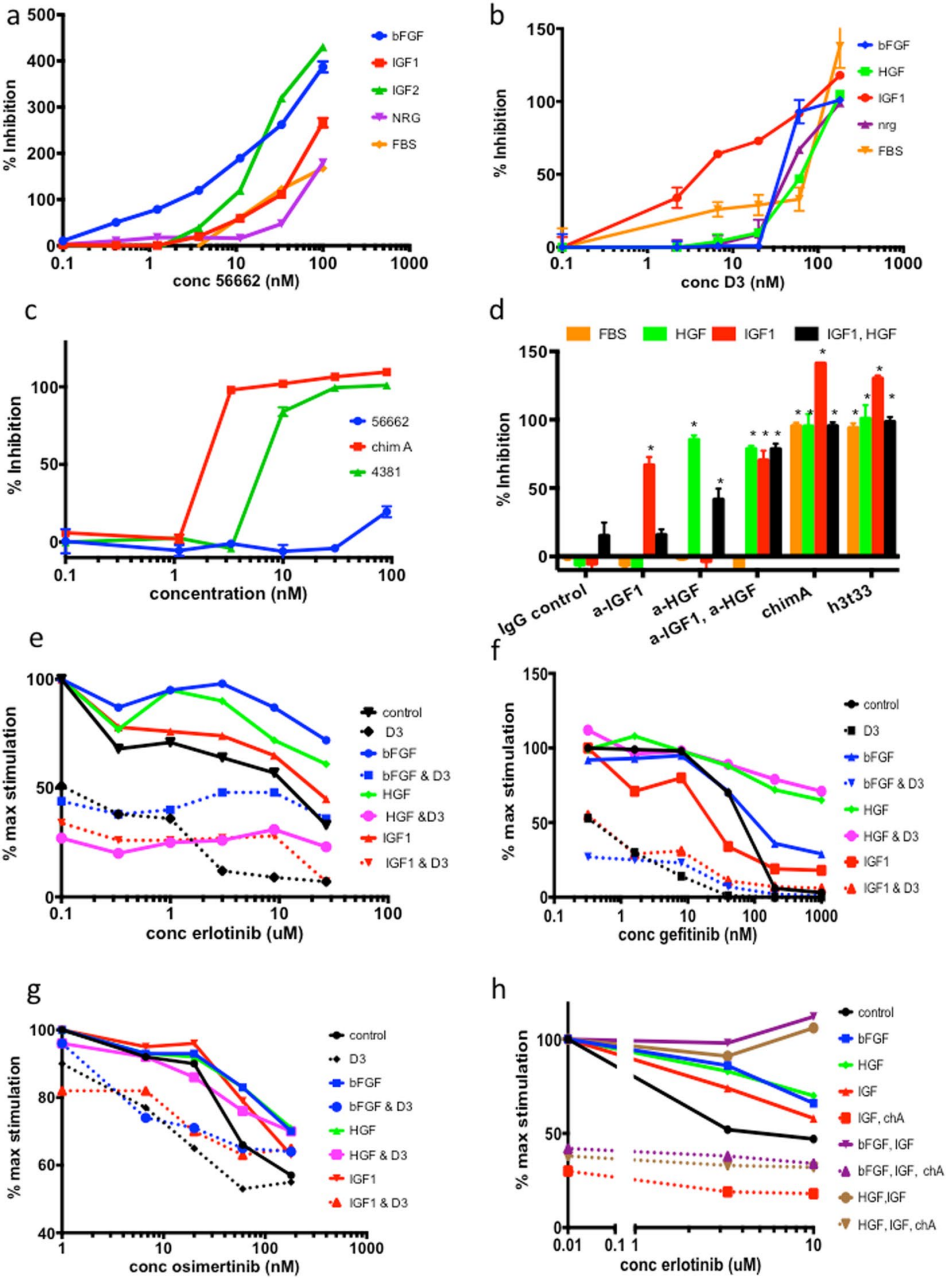
BP3-Fc constructs inhibit growth factor induced proliferation and augment EGFR TKI inhibition. Percent inhibition is calculated as (maximum stimulated value – observed value)/(maximum stimulated value – unstimulated value); therefore >100% inhibition indicates proliferation below the unstimulated baseline (no added growth factor). Percentage maximum stimulation is calculated as (observed value/value of no drug control); growth factors may increase proliferation up to 25% when added to FBS so each condition is normalized. The standard deviation of 2–3 replicate points is shown in panels a–d; s.d. in panels e–h were similar to panels a–d but error bars were omitted to improve clarity. Significant growth factor rescues for panes e–h are summarized in Supplementary Table S4. In panels a–c no BP3-Fc (0% inhibition) is plotted at 0.1 nM construct because of log-transformation; in panels e-h the zero-drug value (100% max stim) is plotted similarly. In panels a-c the lowest significant inhibitory concentrations are noted in parentheses. (**a**) 56662 inhibits growth factor stimulated proliferation in MCF–7 cells: 0.6 nM bFGF (12.5 nM); 4 nM IGF1 (16.67 nM), 4 nM IGF2 (5.56 nM); 1 nM NRG (12.5 nM); 1% FBS (25 nM). (**b**) D3 inhibits growth factor stimulated proliferation in Hep3B cells: 1 nM bFGF (60 nM); 1 nM HGF (20 nM); 1 nM IGF1 (2.22 nM); 1 nM NRG (60 nM); 1% FBS (60 nM). (**c**) Only VEGF-trap containing contsructs inhibit 1 nM VEGF-induced proliferation in HUVEC cells: 56662 (not significantly different from control); chimera A (3 nM); 4381 (10 nM); IC_50_ values of chimera A and 4381 are not significantly different. (**d**) BP3-Fcs inhibit growth factor combinations in Hep3B cells; significant differences are marked with *: 1% FBS, 0.4 nM HGF, 1 nM IGF1; 8 ug/ml anti-HGF, 2 ug/ml anti-IGF1, 200 nM chimera A, 200 nM h3t33. (**e**) 150 nM D3 augments erlotinib response and eliminates growth factor rescue in Hep3B cells: 0.5 nM bFGF, 0.5 nM HGF, 1 nM IGF1. (**f**) 150 nM D3 augments gefitinib response and inhibits growth factor rescue in PC-9 cells: 0.5 nM bFGF, 0.3 nM HGF, 1 nM IGF1. (**g**) 200 nM D3 augments osimertinib response in H1975 cells and inhibits growth factor rescue: 0.2 nM, 0.2 nM HGF, 2 nM IGF1. (**h**) A combination of IGF1 and HGF will completely rescue Hep3B from erlotinib (no FBS); 100 nM chimera A inhibits combination rescue; 1 nM all growth factors.

Having established high affinity growth factor binding, we next studied BP3-Fc construct inhibition of growth factor-induced proliferation, choosing four different cell types for growth factor responsiveness. Representative assays are shown in Fig. 1 and averaged IC_50_ values are shown in Table 2. Figure 1a shows 56662 inhibits IGF1, IGF2, bFGF, NRG, and FBS-induced proliferation in MCF-7 cells; similar inhibition is seen with D3 in Hep3B cells in Fig. 1b. Table 2 summarizes results, namely, BP3-Fc inhibits proliferation induced by all its ligands. The IC_50_ of D3 versus 56662 is significantly reduced in assays of Hep3B cells stimulated by FBS and IGF1 and is similar or lower in all other assays (Table 2): we attribute the higher efficacy to the increased stability of D3 (see Supplementary Table S2) since growth factor binding constants are comparable. 56662 inhibits all growth factor stimulated MCF-7 proliferation to a level below that observed in the absence of any stimulant, hence the >100% inhibition (Fig. 1a): BP3-Fc sequestration of endogenous as well as exogenous growth factors and IGF-independent effects may contribute to the observed inhibition.

**Table 2:** IC_50_ Values of BP3-Fc IC_50_ values in nM (+/− s.d.) Significant differences between values are noted by symbols.

Assay line	Hep3B			MCF-7			3T3		HUVEC			
construct	IGFBP-3	56662	D3	IGFBP-3	56662	D3	56662	D3	56662	D3	chA	4381
**stimulus**												
FBS	216+/−14	172+/−26*	91+/−27*	282+/−32	189 + −95	110+/−30						
bFGF	296	74+/−35	59+/−14		111+/−31	26+/−23			61+/−16	39+/−14	54+/−23	no inh
HGF	71+/−21	60+/−28	58+/−22						112	56+/−16	68+/−8	no inh
IGF1	80+/−31#@	42+/−16#*	21+/−8@*	76+/−35	52+/−51	29+/−15				7.45+/−1.3	10.7+/−2.3	no inh
NRG	51+/−21	45+/−21	43+/−10		113+/−69	112+/−69						
PDGF BB							81+/−52	30+/−25				
VEGF									310+/−142*	75+/−22*#&	3.9+/−4.8#	5.9+/−2.1&

The absence of s.d. indicates a calcluation based on a single assay.

The VEGF-trap containing constructs - Chimera A and the VEGF-trap control 4381 - inhibit VEGF-induced proliferation. High concentrations of 56662 inhibit VEGF-stimulated proliferation slightly (Fig. 1c), and this effect is likely caused by sequestration of growth factors present in the FBS in the assay. Constructs containing the BP3 domain can inhibit IGF1 or HGF stimulated proliferation in HUVECs (Table 2), but chimera A only can completely inhibit proliferation on HUVECs stimulated by a combination of VEGF and HGF (Supplementary Fig. S2).

BP3-Fc also inhibits proliferation stimulated by a combination of growth factors. In Fig. 1d, h3t33 and chimera A completely inhibit proliferation stimulated by a combination of IGFI and HGF in Hep3B cells. Both constructs also inhibit the proliferation induced by 1% fetal bovine serum, whereas both IGF1 and HGF antibodies are required to inhibit the IGF1/HGF combination. Furthermore, the combination of these two antibodies has no effect on FBS-induced proliferation.

### BP3-Fc inhibits growth factor receptor phosphorylation

**Figure 2:**
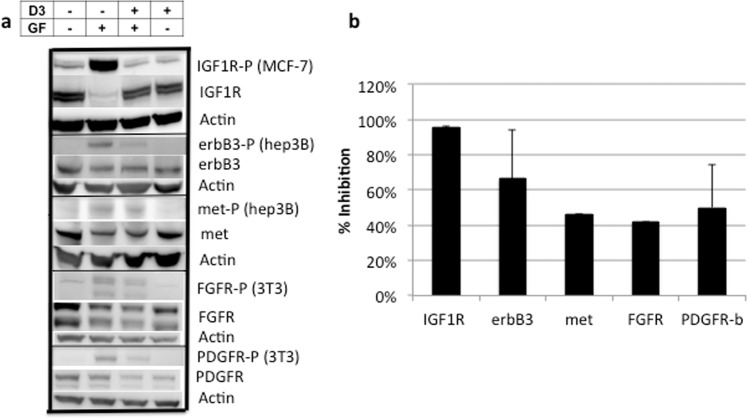
D3 Inhibits Growth Factor Receptor Phosphorylation. Bands were quantitated using Image Studio Lite by Li-Cor, and phosphorylated receptor bands were normalized to non-phosphorylated receptor or actin. a. Representative western blots b. The average of two or more experiments (+/− standard deviation) are summarized in panel b.

D3 inhibition of growth factor stimulated receptor phosphorylation validates growth factor binding as a mechanism of D3 inhibition of proliferation. Figure 2 demonstrates D3 inhibition of growth factor stimulated phosphorylation of the IGF1 receptor, erbB3, Met (HGF receptor), the FGF receptor, and the PDGF receptor β and summarizes results in two or more experiments. The ten-fold higher affinity of D3 for IGF1 compared to its other ligands translates into almost complete inhibition of phosphorylation of IGF1R and significant but incomplete inhibition of the other receptors.

### BP3-Fc increases the efficacy of targeted TKIs on cancer cells

Targeted therapy can cause remarkable cancer regression, but resistance to treatment inevitably develops. Pre-existing mutant cells may become unmasked after drug treatment or resistant cells may evolve from the small percentage of cancer cells that survive treatment with inhibitors; these cells are known as drug-tolerant persisters^31^. These persister cells acquire additional mutations in the target gene or other proliferation pathways^31–34^; thus, the reduction of persisters will likely postpone the development of resistance. Growth factors can rescue cancer cells from targeted therapy, increase the number of persisters, and thus promote resistance^4^. Drug treatment itself can cause an acute cell response that includes growth factor upregulation, and tumor and stromal cells are both sources of growth factors^35,36^.

Basic FGF, HGF, IGF, and NRG can rescue cancer cells from the EGFR TKI erlotinib^4^, so multi-target BP3-Fc constructs may inhibit growth factor rescue by all four growth factors and improve the efficacy of EGFR inhibitors. We examined the effects of D3 with EGFR inhibitors on Hep3B, a hepatoma with wild-type EGFR^37^; PC-9 cells, a human non-small cell lung cancer (NSCLC) line with a EGFR activating exon 19 mutation^38^; and H1975, a NSCLC with EGFR L858R T790M mutations^39^.

### D3 shows greatest utility as an adjuvant to existing therapeutics

In a 3–4 day cell proliferation assay D3 by itself inhibits Hep3B, PC-9, and H1975 cell growth in FBS by 50%, 40%, and 10% (Fig. 1e–g); both growth factor dependent and growth factor independent effects may contribute to D3 activity in Hep3B and PC-9 cells. In Hep3B cells the combination of erlotinib and D3 is highly synergistic (see Supplementary Fig. S3 for isobolograms^40^): in the presence of 10 uM erlotinib 70% of Hep3B cells survive, but D3 addition to 10 uM erlotinib reduces surviving cells to 10% (Fig. 1e); notably, its addition also significantly decreases the IC_50_ of erlotinib by 30-fold (see Fig. 1e and Table 3). This decrease suggests that binding of growth factors endogenously expressed in Hep3B cells^37^ or from FBS increases sensitivity to EGFR inhibition and contributes to the synergism. D3 reduction of AKT phosphorylation only in the presence of erlotinib supports this mechanism. Erlotinib reduces phosphorylation in EGFR and ERK to very low levels, and addition of D3 does not further reduce phosphorylation (Supplementary Fig. S4). Since >10x higher concentrations of erlotinib are required to inhibit wild type EGFR versus exon 19 mutant EGFR^41^, D3 reduction of the IC_50_ value of erlotinib in Hep3B to within observed patient serum concentrations^42^ suggests promising, new therapeutic indications for erlotinib-D3 combination in cancers with wild type EGFR.

**Table 3:** D3 decreases the IC_50_ values of EGFR TKIs.

cell line	drug	IC50 (nM)	IC50 with D3 (nM)	p-value	decrease in IC50 with D3
Hep3B	erlotinib	4378+/−753	205+/−111	<0.0001	28+/−21
PC-9	gefitinib	33+/−10	5+/−3	0.0065	10+/−6
H1975	osimertinib	33+/−9	8+/−3	0.0069	4.6+/−0.9
PC-9IR	gefitinib	1714+/−490	30+/−14	0.0192	71+/−22

PC-9 and H1975 cells are highly sensitive to gefitinib and osimertinib (reported IC_50_ values are 23 and 11 nM, respectively^43^), and the addition of D3 to these drugs at therapeutic concentrations of 1000 and 200 nM has insignificant effects on cell viability in the 3–4 day proliferation assay (Fig. 1f,g). There are modest but significant decreases in the IC_50_ values with addition of D3 to gefitinib in PC-9 (10-fold) and osimertinib in H1975 (5-fold) (Fig. 1f,g and Table 3) which likely reflect increased sensitivity to EGFR inhibition with reduced availability of growth factors.

Basic FGF, HGF and IGF1 partially rescue cells from the EGFR TKIs in all three cell lines as shown in Fig. 1e–h, and a combination of IGF1 and bFGF or IGF1 and HGF will fully rescue Hep3B cells from 10 uM erlotinib (Fig. 1h). Growth factor rescue is significant for bFGF, HGF, and IGF1 for the experiments in these panels except for IGF1 on Hep3B cells in Fig. 1e (see statistics in Supplementary Table S4). In this case, the presence of IGF1 in FBS-containing media likely minimizes the effect of IGF1 addition, since in the absence of FBS, IGF1 rescue of Hep3B cells from erlotinib is significant (Fig. 1h, Supplementary Table S4). D3 completely eliminates bFGF, HGF, and IGF1 and combination rescue in Hep3B cells (Fig. 1e,h). D3 also eliminates bFGF and IGF1 rescue in PC-9, although D3 has only a marginal effect on HGF rescue in PC-9 and H1975 cells (Fig. 1f,g). Although D3 reduces bFGF and IGF1 rescue in H1975, these differences are not significant at the highest concentration of TKI. Supplementary Table S4 summarizes D3 and growth factor effects in Fig. 1e–h and includes p-values for growth factor rescue and D3 inhibition of rescue.

**Figure 3:**
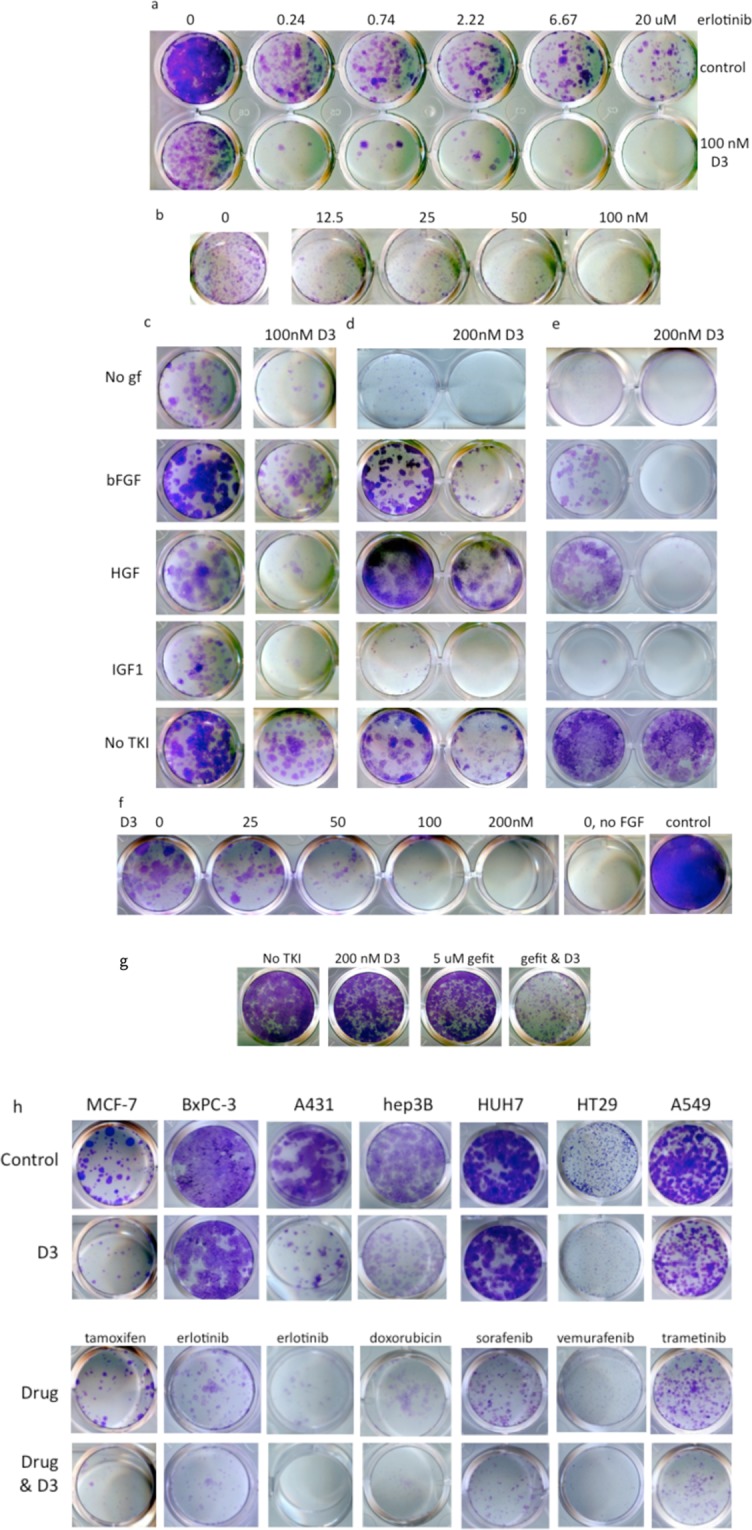
D3 Potentiates EGFR TKI and Inhibits Growth Factor Rescue. (**a**) 1000 Hep3B cells were plated with 100 nM D3 and varying concentrations of erlotinib and grown for 9 days. (**b**) 3000 Hep3B cells were plated with 1 uM erlotinib and varying concentrations of D3 and grown for 9 days. (**c**) 1000 Hep3B cells/well were grown with 10 uM erlotinib for 9 days: no GF, 0.5 nM bFGF, 0.5 nM HGF, 1 nM IGF1. (**d**) 200 PC-9 cells/well were grown for 14 days with 0.25 uM gefitinib except no gf @10,000 cells/well, 2 uM gefitinib: 1 nM bFGF, 1 nM HGF, 1 nM IGF1. (**e**) H1975 cells/well grown with 0.2 uM osimertinib for 14 days: no gf, 32,000 cells/well; 0.25 nM bFGF, 2000 cells/well; 0.25 nM HGF, 1 nM IGF1, no TKI, 500 cells/well. (**f**) 300 PC-9 cells per well were grown with 0. 5uM gefitinib and 0.25 nM bFGF for 14 days and treated with varying concentrations of D3 except no FGF (0.5 uM gefitinib) and control (no addition). (**g**) 1000 PC-9IR cells/well grown for 10 days: 5 uM gefitinib, 200 nM D3. (**h**) Cells were treated with 200 nM D3 and drugs as indicated: MCF-7 (breast, estrogen sensitive), 2.5 uM tamoxifen; BxPC-3 (pancreas), 2.5 uM erlotinib; A431 (head and neck, overexpressed EGFR), 1 uM erlotinib; Hep3B (liver), 10 nM doxorubicin; HUH7 (liver), 2 uM sorafenib; HT29 (colon, BRAFV600E), 1 uM vemurafenib; A549 (lung, *kras)*, 30 nM trametinib.

Growing cells at clonal density allows analysis of the small percentage of persisters surviving inhibitor treatment. The effect seen with D3 and erlotinib in Hep3B cells in the 4-day proliferation assay is replicated in the clonal assay: 100 nM D3 inhibits growth, and D3 with 0.74 uM erlotinib plus 100 nM D3 is more efficacious than even 20 uM erlotinib (Fig. 3a). In the presence of 1uM erlotinib, D3 inhibits growth at a concentration as low as 12.5 nM (Fig. 3b). The clonal assay also reproduces the growth factor rescue of erlotinib-treated Hep3B cells by bFGF, HGF, and IGF1 and the inhibition of growth factor rescue by D3 (Fig. 3c).

Approximately 0.4–4% of PC-9 cells (number of colonies/total cells plated; a colony is defined as >24 cells) survive 14 day treatment with 2 μM gefitinib. The addition of 200 nM D3 to 0.25 uM gefitinib massively decreases both the number of colonies (3 colonies versus 400) and the number of cells per colony (25–32) versus 24- > 700), a > 500-fold reduction in total cells surviving (Fig. 3d, Supplementary Fig. S5). A similar reduction in persister colonies by 200 nM D3 is shown macroscopically and microscopically in PC-9 cells treated with the 2 uM erlotinib (Supplementary Fig. S6). The frequency of surviving colonies varies with plating density, TKI concentration, duration of culture, and characterization of colonies (see Supplementary Figs. S6 and S7 for an examples). Thus D3 clearly reduces persister cell survival, and these clonal assays present aspects of proliferation undetectable in the 4-day survival assay (Fig. 1f).

Consistent with the 4-day proliferation assay, bFGF and HGF, and, to a lesser extent, IGF1 rescue PC-9 cells from gefitinib (Fig. 3d, Supplementary Fig. S7). Basic FGF and HGF not only increase cell survival as indicated by colony number in 0.25 uM gefitinib, but also shift the persisters out of a quiescent state to increase the proliferation rate as indicated by colony size, which is similar to the untreated control. D3 eliminates IGF1 rescue and substantially reduces bFGF and HGF rescue (Fig. 3d), in contrast to D3’s marginal effect on HGF rescue in the 4-day proliferation assay. D3 efficacy of growth factor rescue varies with concentrations of inhibitor, growth factor, and D3: 100 nM D3 almost completely inhibits 0.25 nM bFGF rescue in the presence of 0.5 uM gefitinb (Fig. 3f) in contrast to incomplete inhibition of 1 nM bFGF in with 0.25 uM gefitinib and 200 nM D3 (Fig. 3d.) Similar results with D3 and growth factor rescue in PC-9 cells were seen with 0.2 uM osimertinib (Supplementary Fig. S8) except that 10–40x fewer persister colonies are observed.

Very few H1975 cells (0.01–0.1%) survive 0.2 uM osimertinib treatment after 14 days in culture (Fig. 3e), in contrast to the 4 day proliferation assay which shows 50% cell survival at 180 nM osimertinib (Fig. 1g), suggesting a time requirement for drug dependent killing. Numerous small colonies are seen on day 14 at a plating density of 32,000 cells/well; however, no colony growth is seen with the D3-osimertinib combination (Fig. 3e). IGF1 slightly increases colony number in osimertinib-treated H1975; bFGF and HGF increase survival and colony growth; and addition of D3 substantially reduces these growth factor rescues. Altogether, results in three cell lines with three EGFR TKIs demonstrate that D3 greatly improves the efficacy of existing cancer therapeutics and furthermore reduces resistant rescue by multiple growth factors.

### Addition of D3 restores gefitinib sensitivity to a gefitinib-resistant non-small cell cancer line

**Figure 4:**
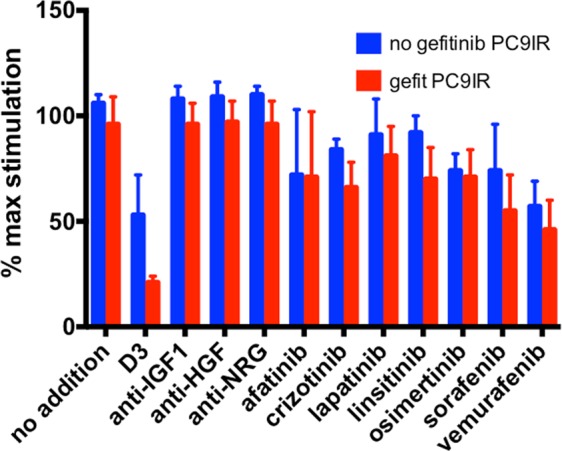
D3 restores sensitivity to gefitinib in PC-9IR cells. Cells were cultured for 4 days in the presence or absence of 1 uM gefitinib. Concentrations of inhibitors: 200 nM D3; 8, 2, 20 ug/ml antibodies; 0.1, 1, 2, 1, 0.5, 2 and 10 uM TKIs. Values and significant differences between treated and control are summarized in Supplementary Table S5.

PC9-IR is a gefitinib resistant clone derived from PC-9 that has undergone epithelial to mesenchymal transition (EMT) and retains the original EGFR mutation^44^. Remarkably, D3 addition reduces the IC_50_ value of gefitinib in PC-9IR cells from 1700 to 30 nM, thus restoring gefitinib sensitivity (Table 3). D3 and gefitinib are highly synergistic in PC-9IR, while the combination is likely additive in the parental PC-9 (Supplementary Fig. S3). Of 11 inhibitors tested, multi-target D3 is the most effective inhibitor in the presence of gefitinib (Fig. 4). The significance of these results is summarized in Supplementary Table S5. Adding D3 to gefitinib also inhibits persister colony formation (Fig. 3g). Our results suggest the combination may prevent EMT and maintain EGFR TKI sensitivity even after EMT.

### D3 potentiates many other cancers and drug treatments

While D3 significantly improves efficacy of EGFR TKIs, it also augments the efficacy of other drugs across a wide range of cancer types. Indeed, D3-inhibitor combinations reduce persister frequency in nine of eleven human cancer cell lines surveyed - all eight *kras* wild type lines tested and one mutated *kras* line (A549). Figure 3h shows that D3 addition reduces the survival of breast, pancreas, skin, liver, colorectal, and lung cancer cells treated with therapeutically achievable concentrations of tamoxifen, erlotinib, doxorubicin, sorafenib, vemurafenib, and trametinib (See Supplementary Figs. 9–11 for additional examples). D3 augmentation of inhibitor activity is dependent on both cell line and inhibitor because cancer cells have different profiles of proliferation drivers (oncogenes and bypass signaling pathways): although D3 inhibits MCF-7 cell growth and increases tamoxifen efficacy, it has no effect when combined with lapatinib (Supplementary Fig. S10); the striking D3-erlotinib combination effect in Hep3B cells is not seen in HUH7 (Fig. 3a, Supplementary Figs. S9 and S11). No effect of D3 was seen in the *kras* mutated lines MDA-MB-231 and PLC/PRF5 treated with erlotinib, crizotinib, sorafenib, or trametinib (MDA-MB-231 data in Supplementary Fig. S11). Thus multi-specific D3 may potentiate precision therapy and chemotherapy in a variety of cancers.

### D3 enhances erlotinib-induced tumor inhibition

**Figure 5:**
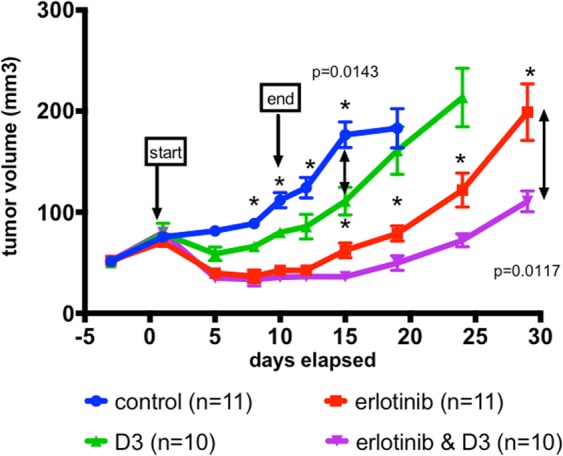
D3 inhibits growth of PC-9 tumors and augments erlotinib efficacy. Five million PC-9 cells were injected subcutaneously in male NOD-SCID mice. D3 was dosed intraperitoneally at 25 mg/kg three times per week; erlotinib was dosed orally daily at 25 mg/kg; control animals were administered vehicle. Erlotinib +/− D3 dosing was started on day 1 and ended on day 10; D3 treatment was discontinued on day 13. Group sizes: control = 11; D3 = 10; erlotinib = 11; erlotinib & D3 = 10. Error bars represent SEM. Statistically significant (p values < 0.05) are marked with *; p value calculations indicated with arrows. Similar results were observed in a repeat study using female NOD-SCID mice.

In the clonal assay D3 alone inhibits FBS-induced proliferation and in combination with a TKI dramatically reduces the persisters (Fig. 3a,c,d) in Hep3B and PC-9 cells. We chose PC-9 cells to examine D3 *in vivo* activity because PC-9 cells exhibited rapid and consistent tumor growth. In Fig. 5 D3 alone at 25 mg/kg inhibited tumor growth modestly from day 5 to day 15. Erlotinib by itself caused tumor regression as expected, and the addition of D3 did not significantly alter tumor volume at the end of treatment (37+/−6 vs. 34+/−6 mm^3^ (SEM)). Erlotinib treatment was stopped after 10 days because of weight loss in erlotinib treated mice. After treatment cessation the D3-erlotinib combination not only delayed tumor regrowth compared to the erlotinib group, but also decreased tumor volume significantly for 20 days (111 mm^3^+/−10 vs. 199 mm^3^+/−28 (SEM)). The approximate 50% reduction in tumor size after 20 days after treatment end in D3 & erlotinib versus erlotinib treatment supports our *in vitro* results that D3 increases tumor cell death in erlotinib treated PC-9 cells.

## Discussion

We have demonstrated that IGFBP-3 can bind bFGF, HGF, NRG, PDGF, and VEGF-B as well as IGF-1 and IGF-2 with high affinity; inhibit proliferation induced by its ligands; and reduce ligand stimulated receptor phosphorylation. IGFBP-3 also binds and inhibits BMP-2^45^ and binds to membrane, cytoplasmic, and nuclear proteins^12,46^. Multi-specific protein-protein interactions are characteristic of signaling proteins (for a review of erbB3, see^47^), and fibronectin^48^ and tenascin^49^ bind members of the FGF, PDGF, TGF-β, and VEGF families with nanomolar affinity, although these interactions do not inhibit the activity of its binding partners. We screened only the growth factors mentioned in results, so IGFBP-3 binding to an untested growth factor may contribute to its complex activities. Our results support growth factor sequestration as a significant contributor to the previously described IGF-independent inhibitory activities of IGFBP-3^13,50^, such as inhibition of bFGF-stimulated proliferation^51^, inhibition of angiogenesis^52^, and tumor suppression^53^. IGFBP-3 also binds to membrane, cytoplasmic, and nuclear proteins^46^, so the involvement of IGFBP3’s other growth factor independent interactions remains to be elucidated.

Drug resistance follows initial response to targeted therapy, and genetically altered resistant cells evolve from drug tolerant persisters^32,33^: thus, persister number may affect the development of resistance. Growth factors rescue TKI treated cells^4^ and also promote the growth of pre-existing mutations that lead to resistance^54^. Since one cannot predict which of multiple growth factors or combinations of growth factors will rescue tumor cells in different cancers, with different drugs, and in individual patients, effective preventative treatment requires combinations of drugs. However, drug combinations may increase adverse effects, so safety remains a significant concern.

We show that a single protein can inhibit multiple growth factor pathways and when combined with an effective cancer drug, reduces the frequency of persister cells and inhibits growth factor rescue at concentrations as low as 12.5 nM. Sequestration of growth factors by D3 allows erlotinib to be effective at therapeutic serum concentrations in Hep3B cells, which possess wild-type EGFR, reducing the IC_50_ values from 4.4 uM to 0.2 uM, so D3 addition may broaden erlotinib therapy in non-mutated EGFR cancers. Furthermore, D3 may reduce intrinsic resistance fueled by elevated growth factor expression^2,55^. The benefits of multiple growth factor inhibition is not limited to EGFR TKIs, and D3 combination with other cancer drugs results in decreased persister frequency in nine out of eleven cancer cell lines tested.

The value of multiple growth factor targeting is also seen in EMT transformed NSCLC. 14% of NSCLC patients who develop erlotinib resistance exhibit EMT, and tumors retain the original EGFR activating mutation^56^. D3 was uniquely active among 10 inhibitors in restoring gefitinib sensitivity in an EMT gefitinib-resistant NSCLC. The success of multiple growth factor inhibition in treating resistance in the absence of a known target suggests the addition of D3 to EGFR TKIs may postpone or inhibit EMT resistance.

Our experiments also show that addition of D3 improves the *in vivo* efficacy of erlotinib. All erlotinib treated PC-9 tumors regressed, but at treatment cessation, resumption of tumor growth was postponed in the D3 combination group so that tumor volume in the D3 plus erlotinib group was approximately 50% smaller than in the erlotinib alone group for 20 days after the end of a 10 day drug treatment. This result supports our hypothesis that D3 addition reduces tumor cell survival *in vivo* and may postpone the development of resistance. While weight loss in all erlotinib treated mice precluded longer dosing, a change in protocol or use of another EGFR TKI may permit us to further extend treatment to examine the development of resistance. While we focused solely on the IGFBP3 binding activity *in vivo* to avoid the additional complexity of VEGF-trap controls, the added anti-angiogenic properties may render BP3-VEGF-trap-Fc even more potent in cancer therapy.

We expect D3 to have low toxicity based on (1) no weight loss or deaths in D3-treated mice compared to controls; (2) the health and fertility of IGFBP-3 overexpressing transgenic mice^57^; and (3) approval of a recombinant IGFBP3 and IGF1 complex (mecasermin rinfabate) for IGF1 deficiency in children^58^. The most common adverse effect of mecasermin is mild hypoglycemia caused by IGF1.

## Conclusion

We discovered that IGFBP-3 can bind bFGF, HGF, NRG, and PDGF as well as IGF1 and IGF2, engineered multiple constructs with improved drug properties which can inhibit the activities of these growth factors, and demonstrated improvement in efficacy when used in combination with numerous clinical standard of care cancer therapeutics in multiple tumor cell lines. The combination of D3 with EGFR TKIs decreases cell survival, inhibits growth factor rescue, and in Hep3B cells with wild type EGFR, reduces erlotinib IC_50_ values to achievable therapeutic concentrations. Furthermore, D3 restores gefitinib sensitivity in a NSCLC cell line that has undergone EMT. Our results suggest that sequestration of multiple growth factors by a single agent, which, in unmodified form, has been approved for clinical use, may improve cancer drug efficacy and postpone the development of resistant cancer cells across many different indications.

## Methods

### Materials

Information for growth factors, antibodies, and other reagents purchased commercially is listed in Supplementary Table S6. Proteins used in Biacore immobilization were purchased carrier-free. Chemical inhibitors were purchased from Selleck Chemicals.

### Chimera construction

We used NP_000589.2 protein sequence to synthesize DNA for IGFBP-3 constructs. The secretory signal peptide was derived from IGFBP-2. DNA was synthesized by DNA2.0 or ThermoFisher Scientific, and sequences were optimized for human codon usage as directed by the supplier. DNA was inserted into pJ603, a DNA2.0 proprietary mammalian expression vector, from DNA2.0. Sequences are listed in Supplementary Table S3.

### Protein Production

Plasmids were produced in E.coli and purified with NucleoBond Xtra Midi Plus Kit (Machevey-Nagel). 293 T cells were transfected using Mirus Transit using the manufacturer’s suggested protocol. After 24 hrs, medium was changed to Opti-MEM, 0.5% FBS, 2 mM valproic acid, 50 ng/ml [arg3]-IGF1. Conditioned medium was harvested every 2 days. Concentrated conditioned medium was purified by proteinA Sepharose to greater than 95% purity when evaluated by SDS gel electrophoresis. Chimeras were analyzed on Superdex 200 equilibrated in 50 mM sodium succinate, 1 M NaCl, pH 6.0 to assess aggregation, and BP3-Fc used in this study showed less than 10% aggregation.

### Measurement of Protein

Elisas for human Fc and IGF1 binding were used to routinely monitor expression and purification and manufacturer instructions were followed. Grenier high binding Elisa plates were coated with 1 ug/ml goat anti-human Fc antibody (Jackson Immunoresearch) in PBS overnight, and then blocked with Assay Buffer (Invitrogen). Human Fc (Jackson Immunoresearch) was used as standard. Plates were incubated with Fc construct and then with HRP-conjugated donkey anti-human Fc antibody (Jackson Immunoresearch). The HRP substrate, tetramethyl benzidine (Invitrogen) was used for detection. For measurement of IGF binding, constructs were bound to anti-human Fc coated Elsa plates, followed by 20 ng/ml biotinylated IGF1 and then streptavidin-HRP. Total protein was measured with a Micro BCA Kit (ThermoFisher).

### Cell culture and assays

MCF-7, MDA-MB-231, A431, BxPC-3, HT29, PC-9, H1975, Hep3B, HUH7, and PLC/PRF5 were maintained as recommended by ATCC. BxPC-3, Hep3B, MCF-7, and PLC/PRF5 cells were obtained from the NHRI Cell Bank, Taiwan; PC-9IR cells were a gift from P.C. Yang, Academia Sinica; all other cells were a gift from T.L. Hsu, Genome Research Center. Cells were used within eight weeks from thawing. Human umbilical vascular endothelial cells (HUVEC) were obtained from SanBio and maintained in endothelial cell medium (ScienCell) with 10% FBS. For proliferation assays, cells except for HUVEC were seeded at a density of 2000–6000 in 96 wells in assay buffer of DMEM/F12 plus 0.2% BSA and 10 ug/ml transferrin. HUVECs were plated at a density of 5000 cells in poly-L-lysine coated 96 wells in M199 with 1%FBS with HEPES. Growth factors or test chimeras in assay buffer were added at the time of plating. For small molecule inhibitor assays, cells were plated in 0.5% or 1% FBS; inhibitors were added after cells had attached. After 3–4 days of culture, growth was determined using incubating cells with Alamar Blue (InVitrogen) for 4–8 hrs and reading the fluorescence at 544/590 nm.

For clonal assays, 300–32,000 cells per well were plated in 24 well dishes in complete medium; inhibitors were added 16–72 hrs later. Cells were grown for 7–33 days and were fed twice a week. Colonies were fixed in methanol and then stained in Giemsa. Plate images were recorded on an Epson scanner. A colony was defined as >24 cells.

Samples were examined in duplicate or triplicate for cell growth assays; each experiment was repeated at least three times; representative results are shown in graphs and images, and averaged results are used in tables. Standard deviations are shown except where noted.

### Biacore binding

The surface plasmon resonance protocol was developed on a Biacore T100 at the Biosensor Lab at the University of Utah; later studies were performed on a Biacore T200 at the Genomic Research Center at Academia Sinica. Surfaces of IGF binding proteins or Fc fusions were made via chemical immobilization on a CM4 chip using standard amine coupling chemistry. Some very basic growth factors showed high background binding to control surfaces and so were immobilized to CM4 chips^59^.  Excess surface carboxyl activated sites on all four flow cells were blocked using the doubly positively charged species ethylenediamine. Binding experiments were performed in HBS-P (GE Lifesciences). BSA (0.2 mg/ml) or NaCl (0.65 M final concentration) was used to reduce the non-specific binding, which varied with different CM4 lots. Analytes were reconstituted in running buffer. All experiments were performed at 25 °C. 1/150 dilutions of phosphoric acid were used to regenerate surfaces when binding protein was immobilized; 4 M MgCl_2_ followed by 5 M guanidinium chloride was used when growth factors were immobilized. Sensorgrams were analyzed as recommended for 1:1 binding in the Biacore Assay Handbook (GE Lifesciences).

### Receptor phosphorylation western blots

Cells were plated at 80–90% confluence in 6-well plates. After 48 hrs, media was changed to serum-free for 24 hrs. Growth factors and D3 were preincubated for 1 hr and added to cells for 10–20 min. Cell lysis, sample preparation and blotting were performed as recommended by the antibody manufacturer. Chemiluminescent imaging was performed on an ImageQuant LAS 4000, and bands quantitated with ImageStudioLite.

### Xenograft studies

Five million PC-9 cells in a 50% suspension with Matrigel were injected subcutaneously over the flank in 6–8 week old NOD/SCID mice. After tumors reached 50–100 mm^3^, mice were sorted by tumor size to form comparable groups. Erlotinib in 6% captisol (Ligand) was dosed orally daily; D3 was dosed intraperitoneally at 25 mg/kg three times per week; control animals were administered vehicle. Tumor volumes were measured with an electronic microcaliper using the formula (length x width^2^/2) 2–3x per week. Significance was calculated using the student’s two-tailed t-test. Two independent studies were performed. The animal protocol (IACUC_14-01-461) was reviewed and approved by the Institutional Animal Care and Use Committee (IACUC) of the Genomics Research Center, Academia Sinica and experiments were performed in compliance with IACUC guidelines.

### Statistics

We used the two-tailed t-test to determine significance. Standard deviation is used for uncertainty values and error bars except where noted.

## Supplementary Information

Supplementary Information.

Supplementary Information.2
