# Supplementary material for: Multiple Growth Factor Targeting by Engineered Insulin-like Growth Factor Binding Protein-3 Augments EGF Receptor Tyrosine Kinase Inhibitor Efficacy
Source: Sci Rep. 2020 Feb 17;10:2735. doi: 10.1038/s41598-020-59466-6 (PMC7026407; doi:10.1038/s41598-020-59466-6)

Multiple Growth Factor Targeting by Engineered Insulin-like Growth Factor Binding Protein-3 Augments EGF Receptor Tyrosine Kinase Inhibitor Efficacy

Elizabeth A. Wang*[1,] , Wan-Yu Chen[1], and Chi-Huey Wong*[1,2]

[1]Genomics Research Center, Academia Sinica, Taipei, 11529, Taiwan

[2]Department of Chemistry, The Scripps Research Institute, La Jolla, CA, 92037, USA

Corresponding authors: wangelizabee@gmail.com, chwong@gate.sinica.edu.tw.

Supplementary Figure S1: Binding of Neuregulin, HGF, and PDGF AB to IGFBP-2, 3, or 7. Growth factors were dissolved in HBS-P supplemented with 0.2 mg/ml BSA to a concentration of 1.0 mg/ml. The concentrations of analyte injected for neuregulin and HGF are 111 nM and 6 three-fold serial dilutions thereof; PDGF AB concentration is 44 nM and three-fold serial dilutions. Binding responses of HGF IGFBP-3 did not fit well to a 1:1 binding model even with allowances for different maximum surface response levels for different injections. a. Growth factor binding to IGFBP-2, -3, and 7. b. Only IGF1 and IGF2 compete with biotinylated IGF1 (20 ng/ml). c. NRG and not VEGF or activin, bind to 56662 in the presence of 50 nM IGF1.

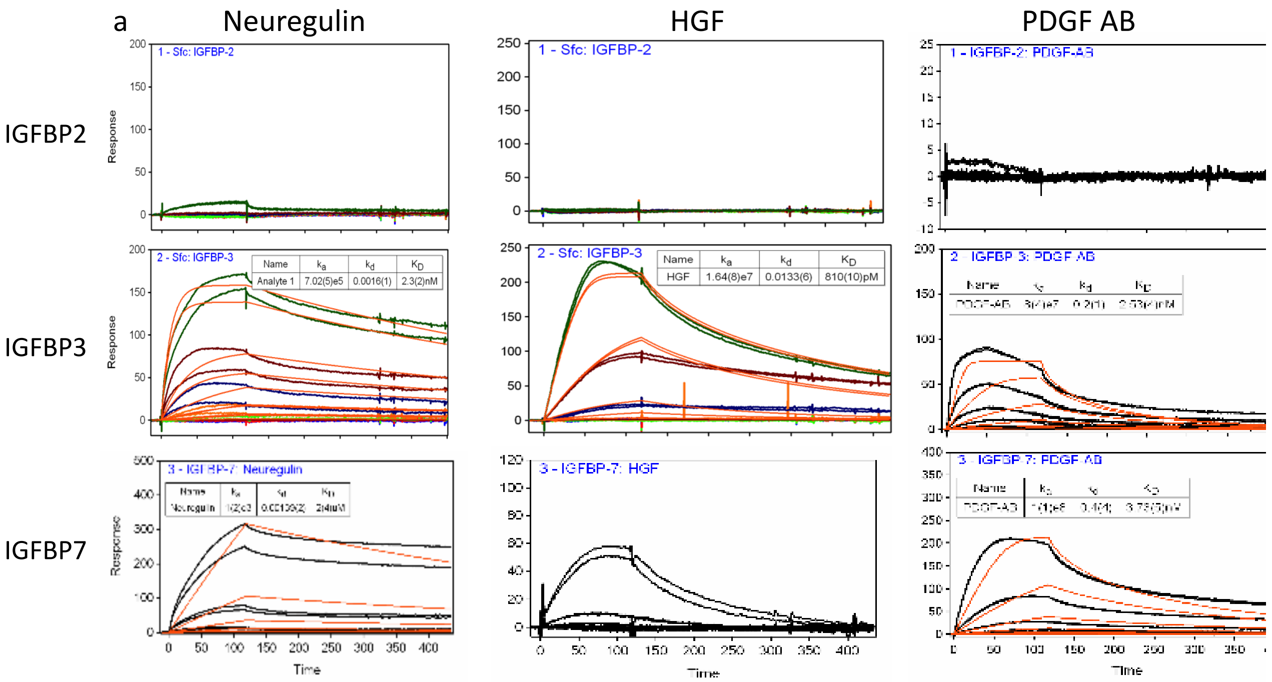

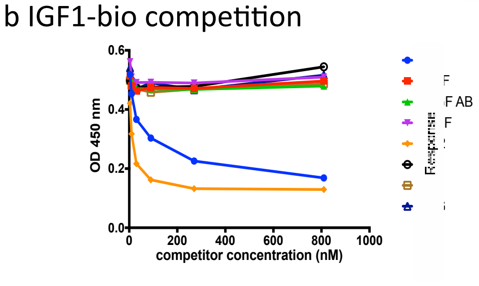

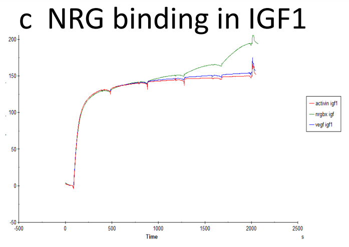

Supplementary Figure S2. Chimera A inhibits HGF and VEGF-induced proliferation of HUVEC.

HUVEC cells were treated with 0.3 nM VEGF, 0.3 nM HGF, or a combination of both and with 30 nM construct for VEGF and 180 nM construct for HGF and HGF-VEGF combination.

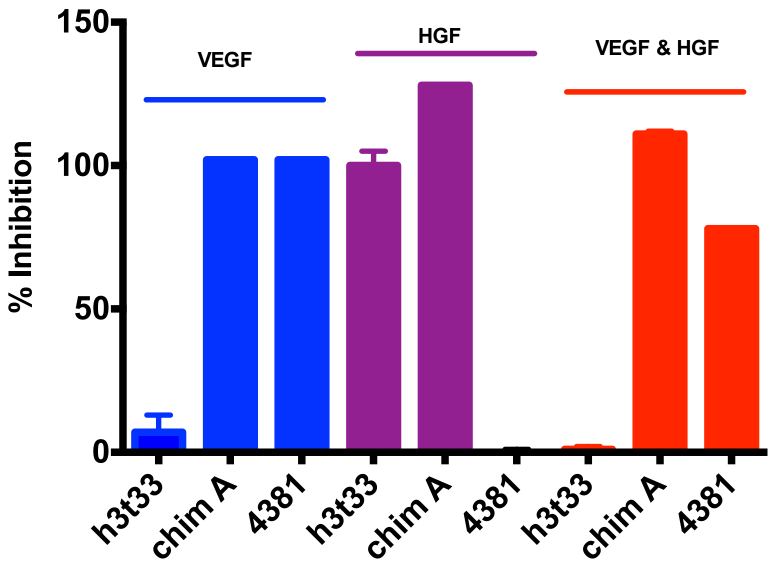

Supplementary Figure S3. Chou-Talalay isobolograms demonstrate synergism of BP3-Fc and EGFR TKIs. a. Isobologram at 40% max stimulation (1% FBS or 4 nM IGF1) of Hep3B cells treated with erlotinib and D3. b. Isobologram at 40% max stimulation (4nM IGF1) of Hep3B cells treated with erlotinib and chimera A. c. Isobologram at 50% max stimulation (1% FBS) of PC-9 cells treated with gefitinib and D3. d. Isobologram at 50% max stimulation (1% FBS) of PC-9IR cells treated with gefitinib and D3. (no data at 0 nM D3 because PC-9IR cells are resistant to gefitinib and % max stimulation is 74% at 5 uM gefitinib, no D3.)

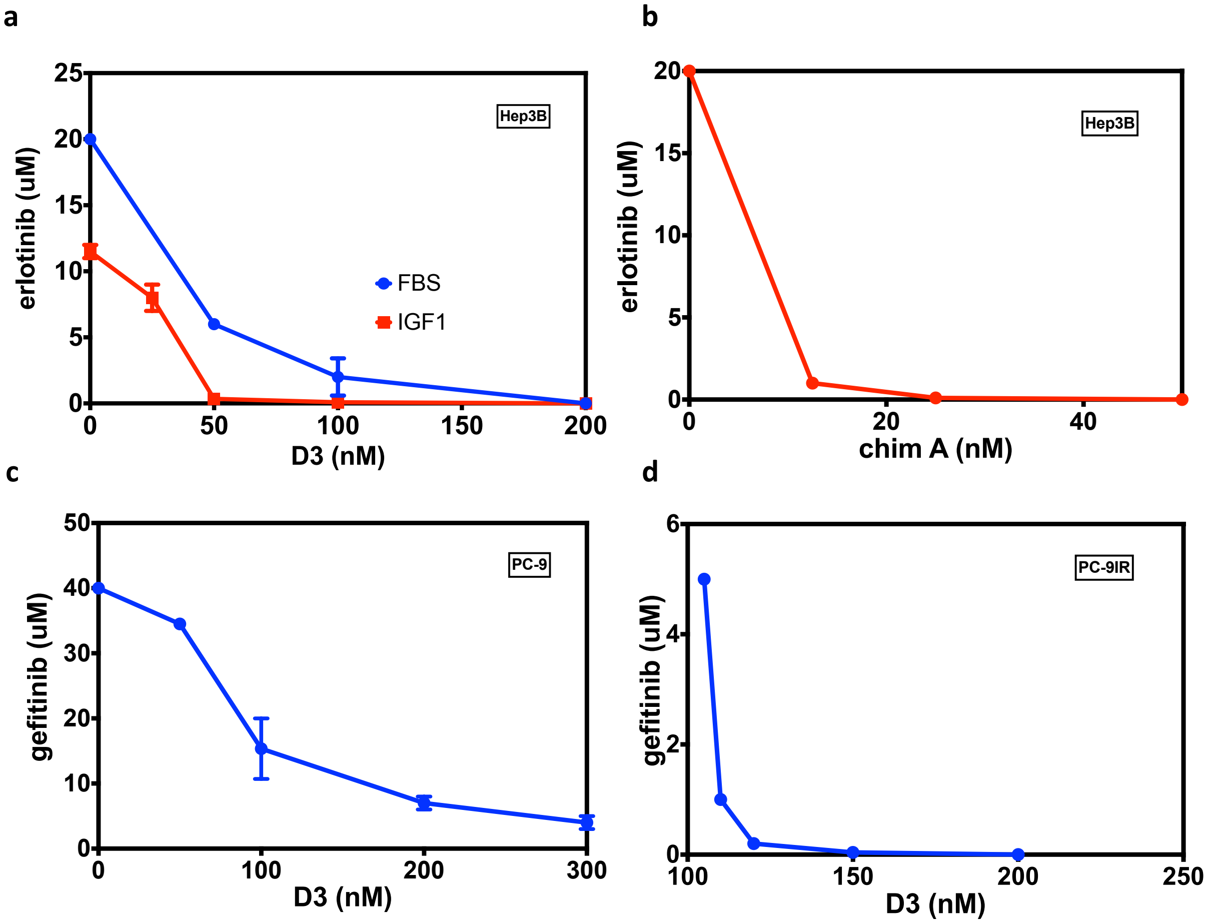

Supplementary Figure S4. D3 reduces AKT phosphorylation in the presence of erlotinib in Hep3B cells. Effector concentrations were 4uM erlotinib, 200nM D3, and 0.5nM EGF.

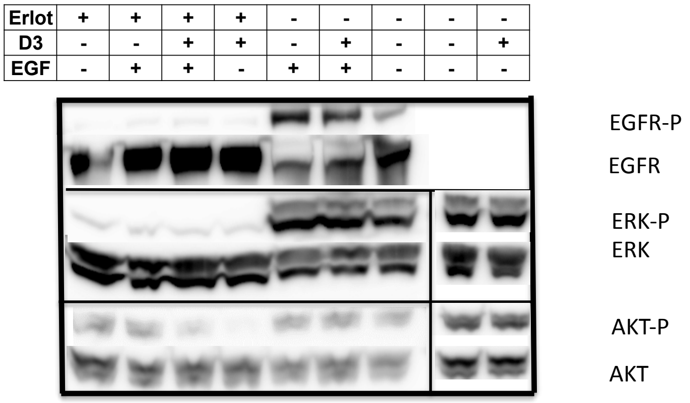

Supplementary Figure S5. D3 reduces persister colonies in PC-9 cells treated with gefitinib. a. 10,000 PC-9 cells were plated and treated as indicated for 14 days. b. Table of estimated colony number and colony size.

a

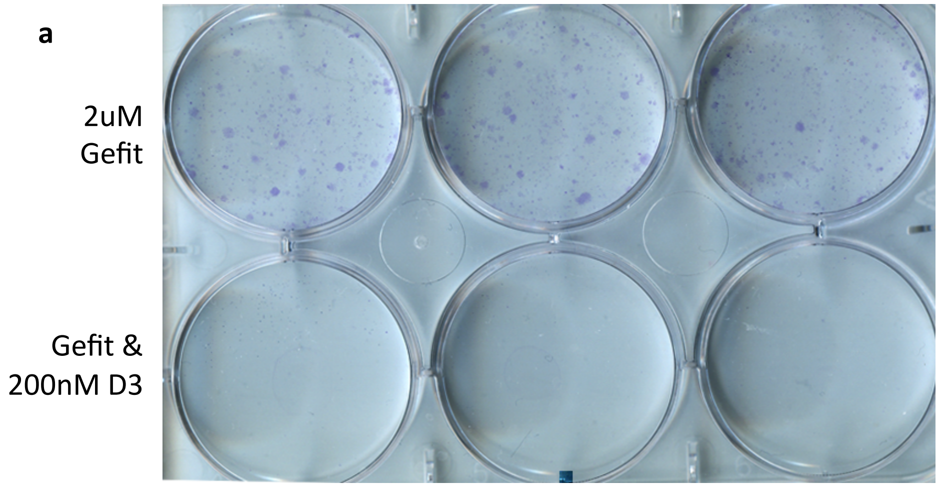

b

PC9	Colonies per well	average size (range)	total cells per well
2uM gefitinib	400	151 (24->700)	60,400
Gefitinib & 200 nM D3	3	28 (25-29)	84

Supplementary Figure S6. D3 reduces persister colonies in PC-9 cells treated with erlotinib. a. 1000 PC-9 cells plated and treated as indicated for 12 days. b. Micrographs of representative areas of wells in panel a (10x magnification).

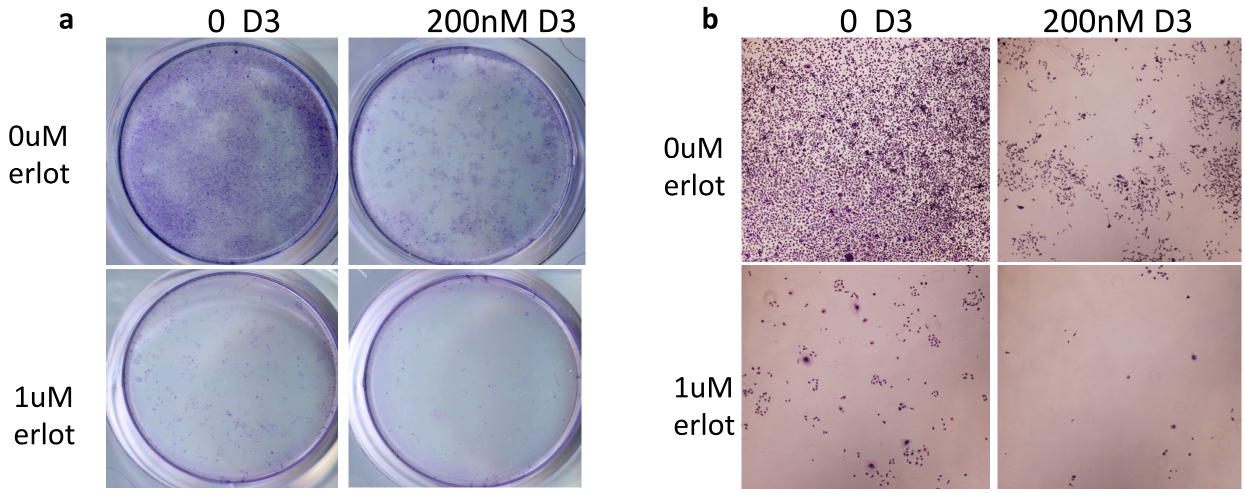

Supplementary Figure S7. D3 reduces persister cell survival and reduces bFGF rescue in PC-9 cells treated with 2 uM gefitinib . Approximate colony counts and colony size range (in parentheses) are shown for controls (2 uM gefitinib). The ~200 visible clusters of cells (8-24) in the control well at 7 days are not considered colonies. No colonies were observed in control or IGF1 wells treated with D3.

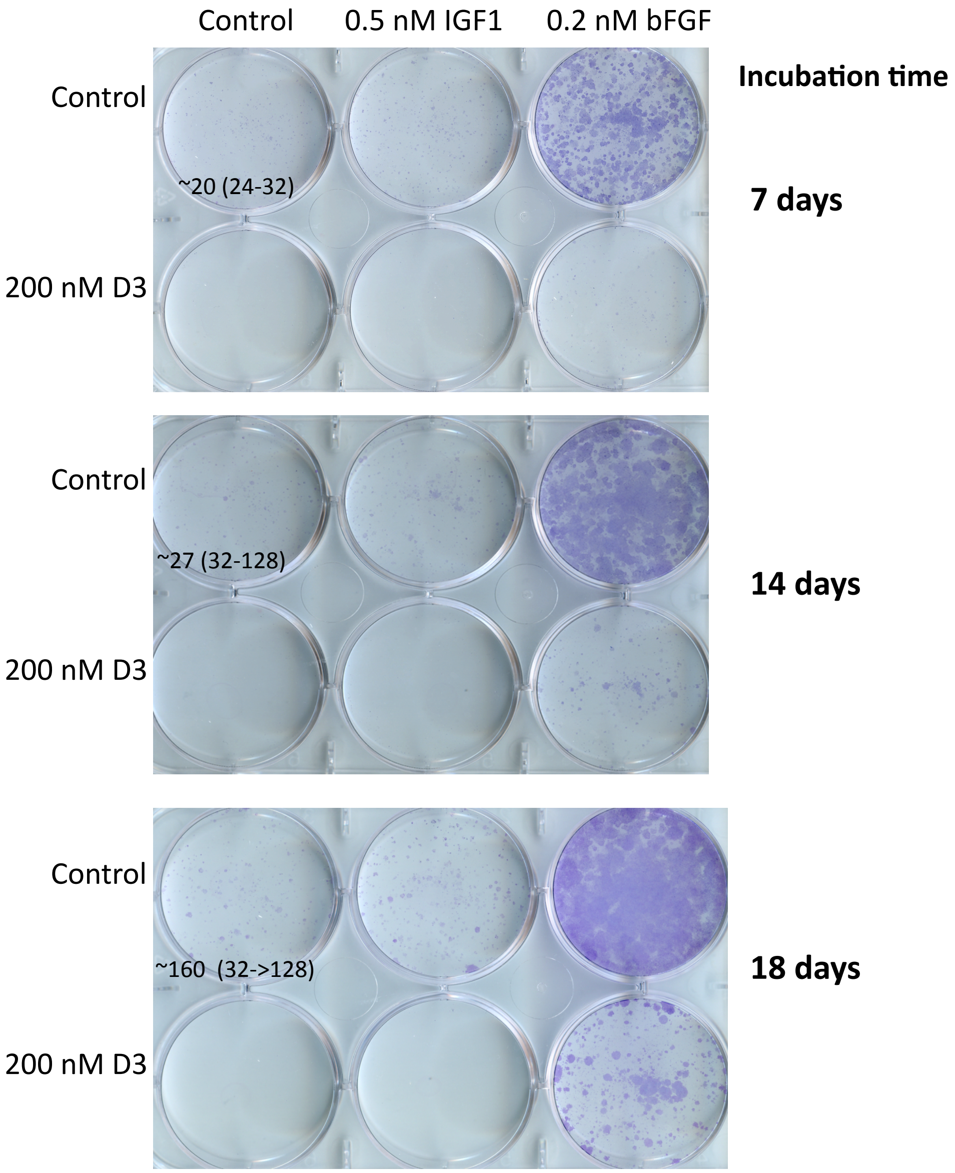

Supplementary Figure S8. D3 reduces cell survival and inhibits GF rescue in osimertinib-treated PC-9 cells. a. 10,000 cells/well were grown with 2 uM gefitinib(a1) or 20,000 cells with 0.2 uM osimertinib (a2). b-e: 1000 cells per well grown with 0.2 uM osimertinib +/- 150 nM D3 b. no growth factor c. 0.25 nM bFGF d. 0.25 nM HGF e. 1.0 nM IGF1 f. no osimertinib

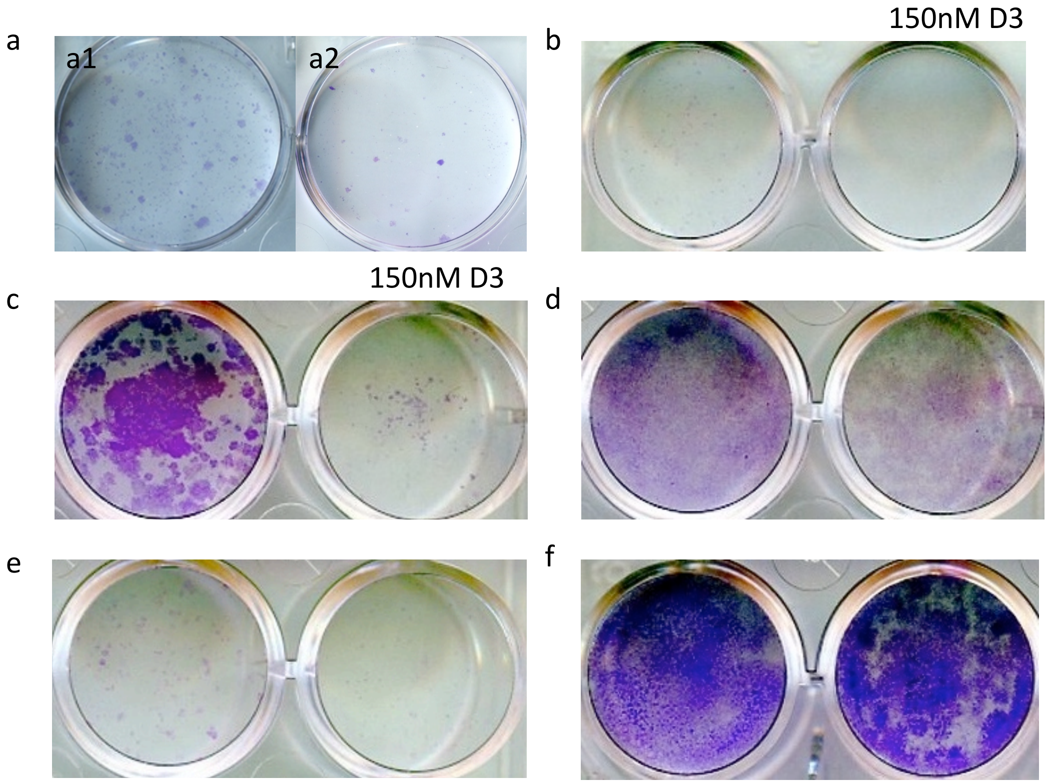

Supplementary Figure S9. D3 augments TKI activity in Hep3B cells. a.1000 Hep3B cells were treated with inhibitors and grown for 14 and 24 days. b. 1000 Hep3B cells were treated as indicated (0.25nM bFGF, 0.25nM HGF, 2nM IGF1) and grown for 12 days.

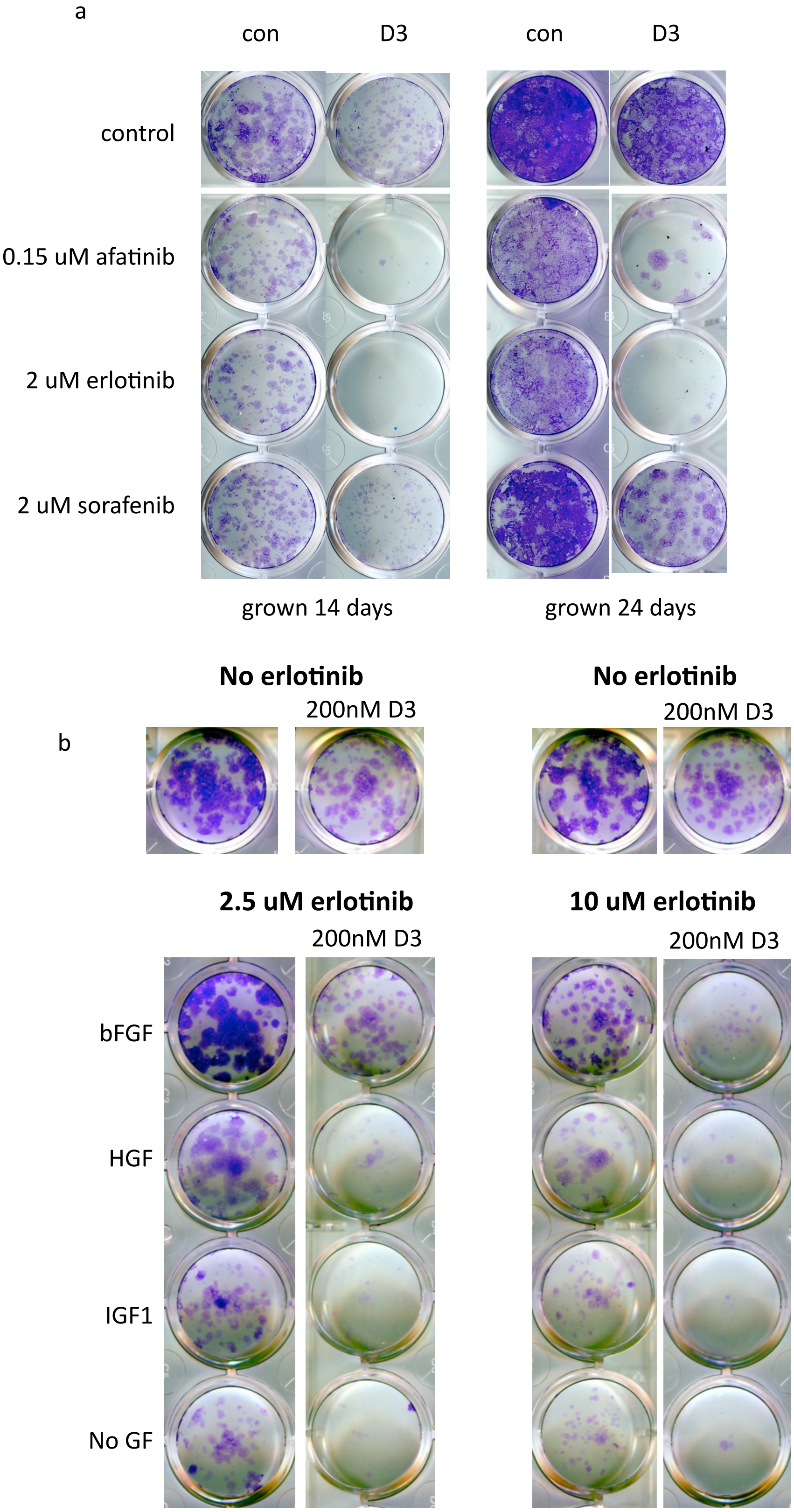

Supplementary Figure S10. D3 and inhibitor activity in MCF-7 and A549 cells. 500 MCF-7 cells were plated and treated as indicated for 14 days. 1000 A549 cells were plated and grown for 9 days.

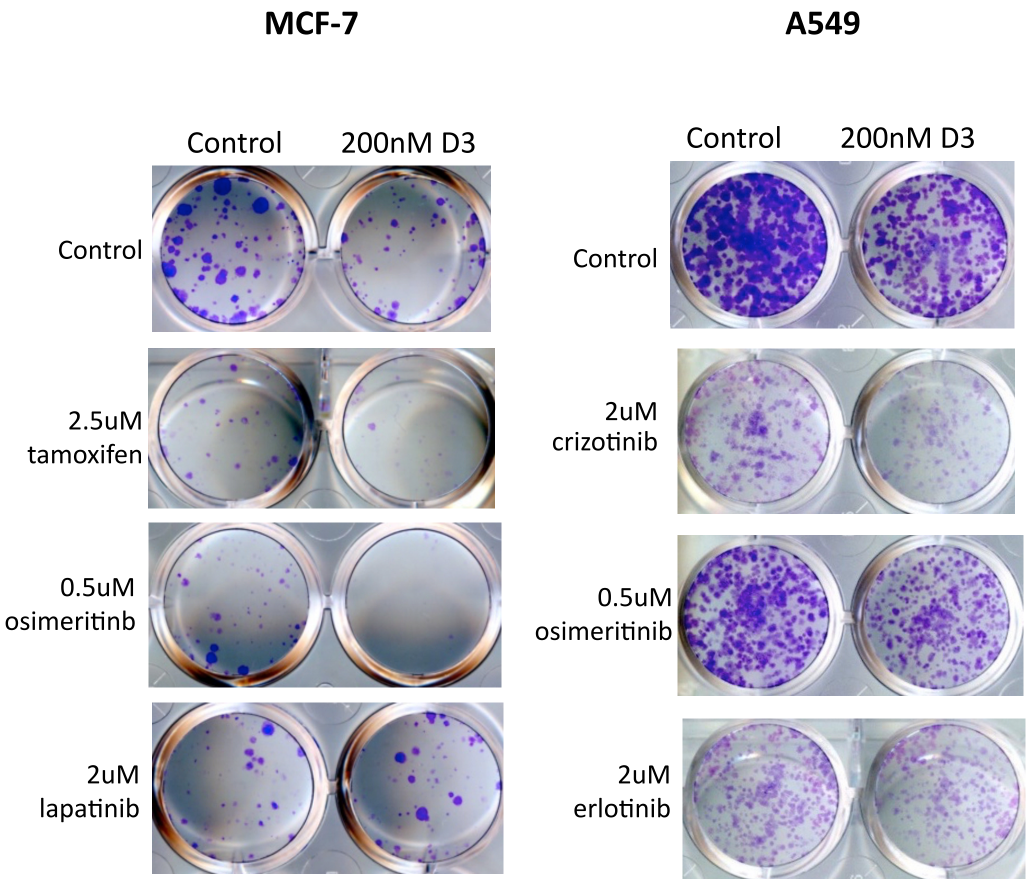

Supplementary Figure S11. Survey of TKIs on HUH7 and MDA-MB-231 cells. 1000 HUH7 cells were plated and grown for 13 days. 1000 MDA-MB-231 cells were plated and grown for 12 days. Drug concentrations used were 1uM, 0.5uM, 2uM, 2uM, 0.5uM, 2 uM, 30nM.

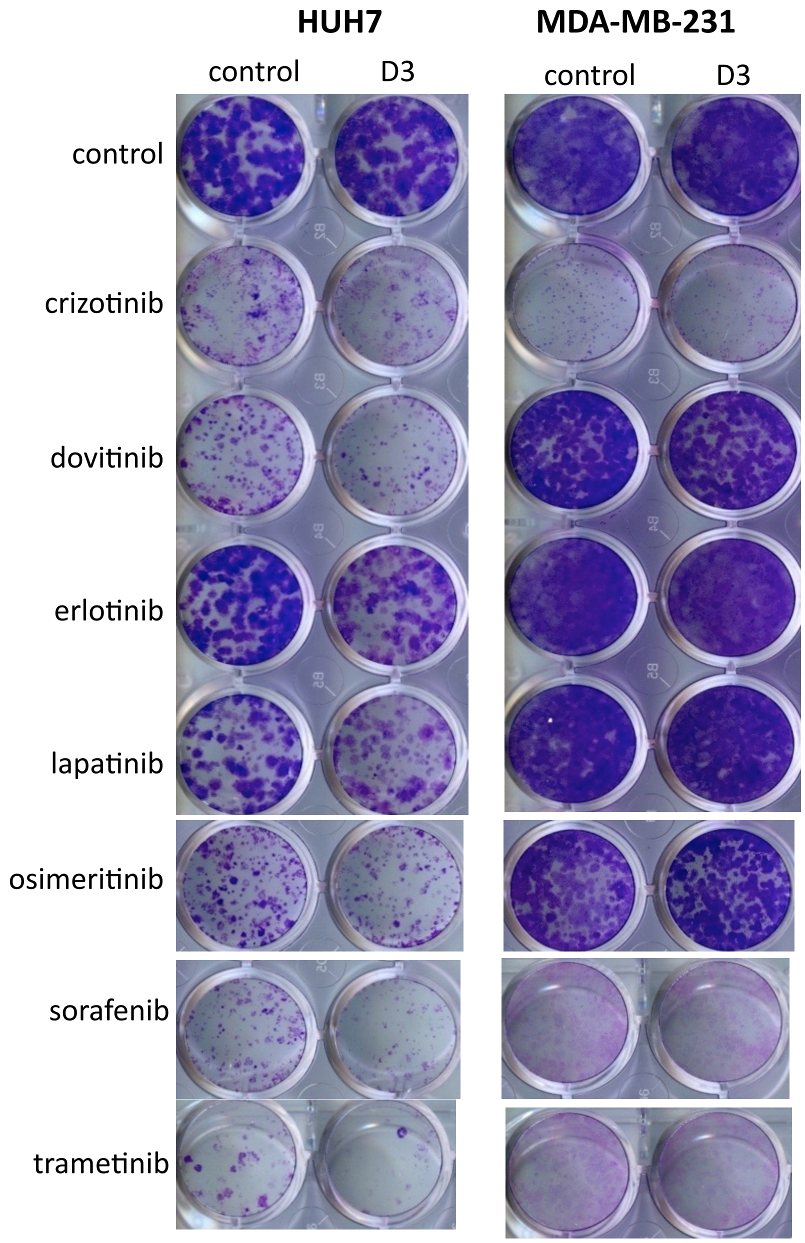

Supplementary Table S1. Association and dissociation constants of IGFBPs and BP3-Fc.
Please see separate Excel file. k_a ($M^{-1}s^{-1}$); k_d (s^{-1}); K_D (M))

Supplementary Table S2. Stability of BP3-Fc constructs
BP3-Fc was incubated at 37 for 0 (control) and 20 hrs in serum-free medium or 10x concentrated medium conditioned for 48 hrs by MCF-7, MDA-MB-231, or HUVEC cells. Fc concentration and IGF1-bio binding was measured by Elisa. Averaged values, standard deviations, and p values compared to 56662 are shown.

% Fc remaining	control	37 control	MCF7	231	HUVEC
56662	100	80 +/- 22	70 +/-30	110 +/-7	126
D3	100	109	102 +/- 10	88 +/-2	90
h3t33	100	97 +/- 12	97 +/-12	96 +/-1	94
% IGF1-bio binding	control	37 control	MCF7	231	HUVEC
56662	100	57 +/- 16	25 +/-3	21 +/-4	47
D3	100	95 +/- 8<br>p=0.0013	58 +/12<br>p=0.004	49 +/- 7<br>p=0.0113	97
h3t33	100	97 +/-6<br>p=0.0014	53 +/- 2<br>p=0.002	56 +/- 1<br>o=0.0267	106

Supplementary Table S3. Amino Acid Sequences of Constructs

We have substituted the signal sequence of IGFBP-3 with that of IGFBP-2. The N-terminus (GASSAGLGPVVR), determined by mass spectroscopy of trypsin digested of 56662, is the same as wildtype IGFBP-3.

56662 (IGFBP-3-Fc)
MSEVPVARVWLVLLLLTVQVGVTAGGASSAGLGPVVRCEPCDARALAQCAPPPAV
CAELVREPGCGCCLTCALSEGQPCGIYTERCGSGLRCQPSPDEARPLQALLDGRGLCVNASAVS
RLRAYLLPAPPAPGNASESEEDRSAGSVESPSVSSTHRVSDPKFHPLHSKIIIIKKGHAKDSQRYKV
DYESQSTDTQNFSSESKRETEYGPCRREMEDTLNHLKFLNVLSPRGVHIPNCDKKGFYKKKQCR
PSKGRKRGFCWCVDKYGQPLPGYTTKGKEDVHCYSMQSKVECPPCPAPPVAGPSVFLFPPKPK
DQLMISRTPEVTCVVVDVSHEDPEVKFNWYVDGVEVHNAKTKPREEQYNSTYRVVSVLTVLHQD
WLNGKEYKCKVSNKGLPSSIEKTISKAKGQPREPQVYTLPPSREEMTKNQVSLTCLVKGFYPSDIA
VEWESNGQPENNYKTTPPVLDSDGSFFLYSKLTVDKSRWQQGNVFSCSVMHEGLHNHYTQKSL
SLSPGK

H3t33 (BP3-deletion-Fc)
56662 with deletion of amino acids 120-174.

D3 (BP3-deletion-Fc)

56662 with deletion of amino acids 121-126; 166-174; 180-185

4381 (VEGF-trap-Fc)
MSEVPVARVWLVLLLLTVQVGVTAGIYIFISDTGRPFVEMYSEIPEIIHMTEGRELVIPCRVTSPNITV
TLKKFPLDTLIPDGKRIIWDSRKGFIISNATYKEIGLLTCEATVNGHLYKTNYLTHRQGYRIYDVVLSP
SHGIELSVGEKLVLNCTARTELNVGIDFNWEYPSSKHQHKKLVNRDLKTQSGSEMKKFLSTLTIDG
VTRSDQGLYTCAASSGLMTKKNSTFVRVHEKVECPPCPAPPVAGPSVFLFPPKPKDQLMISRTPE
VTCVVVDVSHEDPEVKFNWYVDGVEVHNAKTKPREEQYNSTYRVVSVLTVLHQDWLNGKEYKC
KVSNKGLPSSIEKTISKAKGQPREPQVYTLPPSREEMTKNQVSLTCLVKGFYPSDIAVEWESNGQ
PENNYKTTPPVLDSDGSFFLYSKLTVDKSRWQQGNVFSCSVMHEGLHNHYTQKSLSLSPGK

Chimera A (BP3-deletion- linker-VEGF-trap-Fc)
MSEVPVARVWLVLLLLTVQVGVTAGGASSAGLGPVVRCEPCDARALAQCAPPPAVCAELVREPG
CGCCLTCALSEGQPCGIYTERCGSGLRCQPSPDEARPLQALLDGRGLCVNASAVSKGHAKDSQR
YKVDYESQSTDTQNFSSESKRETEYGPCRREMEDTLNHLKFLNVLSPRGVHIPNCDKKGFYKKK
QCRPSKGRKRGFCWCVDKYGQPLPGYTTKGKEDVHCYSMQSKAPPAPGNASESEEDRSAGIYI
FISDTGRPFVEMYSEIPEIIHMTEGRELVIPCRVTSPNITVTLKKFPLDTLIPDGKRIIWDSRKGFIISN
ATYKEIGLLTCEATVNGHLYKTNYLTHRQGYRIYDVVLSPSHGIELSVGEKLVLNCTARTELNVGID
FNWEYPSSKHQHKKLVNRDLKTQSGSEMKKFLSTLTIDGVTRSDQGLYTCAASSGLMTKKNSTFV
RVHEKVECPPCPAPPVAGPSVFLFPPKPKDQLMISRTPEVTCVVVDVSHEDPEVKFNWYVDGVE
VHNAKTKPREEQYNSTYRVVSVLTVLHQDWLNGKEYKCKVSNKGLPSSIEKTISKAKGQPREPQV
YTLPPSREEMTKNQVSLTCLVKGFYPSDIAVEWESNGQPENNYKTTPPVLDSDGSFFLYSKLTVD
KSRWQQGNVFSCSVMHEGLHNHYTQKSLSLSPGK

Supplementary Table S4. D3 inhibition of growth factor rescue in Figure 1e-h.
Tabulation of percentage max stimulation values from Figure 1. [a] p-value for growth factor rescue (no addition and growth factor). [b] p-value for D3 inhibition (D3 vs no D3)

		no added GF		bFGF		HGF		IGF1		bFGF&IGF1		HGF&IGF1	
cell line inhibitor		con	D3	con	D3	con	D3	con	D3	con	D3	con	D3
hep3B, 27uM erlotinib (FBS)	% max stim	57.5	30	87	48.4	72.5	45.7	65	28.3				
	p value [a]			0.0003		0.0109		ns					
	p value [b]		0.0007		0.0007		0.0054		0.0259				
PC-9, 1uM gefitinib (FBS)	% max stim	4.5	4.5	34	3	63	68	29.5	6.5				
	p value [a]			0.0105		0.0028		0.0029					
	p value [b]		ns		0.0092		ns		0.0009				
H1975, 180nM osimeritinib (FBS)	% max stim	57	51	79.5	57	102	95	62.5	50				
	p value [a]			0.0175		0.038		0.0465					
	p value [b]		ns		ns		ns		ns				
		con	chA	con	chA	con	chA	con	chA	con	chA	con	chA
hep3B, 10uM erlotinib (no FBS)	% max stim	47		65.5		70		57	29	112	34	106	32
	p value [a]			0.0036		0.0093		0.0389		0.0091		0.0091	
	p value [b]								0.0028		0.0039		0.004

.

Supplementary Table S5. D3 restores sensitivity to gefitinib in PC-9IR cells. Summary of inhibitor treatments shown in Figure 4 on PC-9IR and PC-9 cells. Significance (p-values) between inhibitor/no addition and gefitinib/no gefitinib are listed for PC-9IR cells; because PC-9 cells are sensitive to gefitinib, only inhibitor/no addition p-values are listed for PC-9 cells.

	PC-9IR				PC-9		
	% max stim		p-values		% max stim		p-values
inhibitor	no gefitinib	1uM gefitinib	inhibitor vs. no addition	gefitinb vs. no gefitinib	no gefitinib	1 uM gefitinib	inhibitor vs. no addition (column 5)
no addition	106% +/- 4	96 +/-13		ns	105 +/-5	5+/-1	
200nM D3	53 +/-19	21 +/-3	0.0044	0.0182	65 +/-22	1 +/-1	0.0202
anti-IGF1	108 +/- 6	96+/-10	ns	ns	98 +/-7	7 +/-2	ns
anti-HGF	109 +/-7	97 +/- 10	ns	ns	105 +/-7	6 +/-2	ns
anti-NRG	110 +/-4	96 +/-11	ns	ns	103 +/-6	6+/-3	ns
afatinib	72 +/-31	71 +/-31	ns	ns	5 +/-2	5 +/-1	<0.0001
crizotinib	84 +/-5	66 +/-12	0.0003	0.028	68 +/-9	3+/-1	0.0191
lapatinib	91 +/-17	81 +/-14	ns	ns	69 +/-6	6 +/-1	0.0121
linsitinib	92 +/-8	70 +/-15	ns	ns	84 +/-10	3 +/-1	ns
osimertinib	74 +/-8	71+/- 13	0.0008	ns	6 +/- 2	5 +/-1	<0.0001
sorafenib	74 +/-22	55 +/-17	0.0128	ns	42 +/- 21	9 +/- 1	0.0014
vemurafenib	57 +/-12	46 +/-14	0.0276	ns	58 +/-28	3 +/-3	ns

Supplementary Table S6: Commercial sources of materials

Catalogue #	R&D Systems
AF-294-SP	Hu HGF antibody
AF-233-NA	Hu FGF basic antibody
AF-396-NA	Hu NRG1-B1/HRG1-B1 EGF domain antibody
AF-291-NA	Hu IGF-1 antibody
AF-321	Hu VEGF R1/Flt1 antibody
BAF294	Hu HGF Biotinylated antibody
BAF675	Hu IGFBP3 Biotinylated antibody
674-B2	rhIGFBP-2
675-B3	rhIGFBP-3
876-B6	rhIGFBP-6
1334-B7	rhIGFBP-7
232-FA	acidic FGF
291-G1	IGF1
292-G1	IGF2
233-FB	bFGF
294-HGN	HGF
377-HB	neuregulin extracellular domain
222-AB	PDGF-AB
220-BB	PDGF-BB
293-VE	VEGF A
751-VE	VEGF-B
2179-VC	VEGF-C
622-VD	VEGF-D
232-FA	acidic FGF
976-CX	rhCXCL16
259-GE	rhHB-EGF
208-IL	rhIL-8
509-MI	rhMIP1a
271-BME	rhMIP1b
264-PG	rhPlGF
	Jackson Immunoresearch
016-030-084	peroxidase-conjugated streptavidin
709-035-098	peroxidase-conjugated affinipure Donkey Anti-Human IgG
705-035-003	peroxidase-conjugated affinipure Donkey Anti-Goat IgG
109-005-098	affinipure Goat Anti-Human IgG
005-000-003	ChromPure Goat IgG

	Abcam
ab84228	anti- IGFBP3 antibody (biotin)
ab9572	Hu IGF-1 antibody
	GroPep
AQU010	Biotinyl-IGF-1
LM-005	Human [Arg3] IGF-I
	Cell Signaling
3476	phospho-FGF Receptor (tyr653/654)(55H2) Mouse mAb
9740	FGF Receptor1(D8E4) Rabbit mAb
4791	phospho-HER3/ErbB3(Tyr1289)(21D3) Rabbit mAb
12708	HER3/ErbB3(D22C5) Rabbit mAb
3126	phospho-Met (Tyr1234/1235) Rabbit mAb
8198	Met (D1C2) Rabbit mAb
3173	phospho-PDGF Receptor b(Tyr771)(76D6) Rabbit mAb
3169	PDGF Receptor b(28E1) Rabbit mAb
3777	phospho-EGF Receptor (tyr1068)(D7A5) Rabbit mAb
4267	EGF Receptor (D38B1) Rabbit mAb
4970	b-actin (13E5) Rabbit mAb
7074	Anti-rabbit, HRP-linked Antibody
7075	Anti-biotin, HRP-linked Antibody
7076P2	Anti-mouse, HRP-linked Antibody
3918	anti-phopho-IGF1 Receptor (tyr 1135)
9750	rabbit anti-IGF1 Receptor (D23H3)
9101	phospho-P44/42 MAPK(Erk1/2)(Thr202/tyr204) Rabbit mAb
9102	P44/42 MAPK(Erk1/2) Rabbit mAb
4691	Akt(pan)(C67E7) Rabbit mAb
4060	phospho-Akt(ser473)(D9E) Rabbit mAb

Supplemental Data: Original Western Blots

Figure 2.
IGF1 receptor

P-igf1r

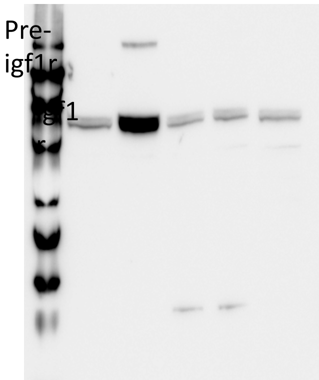

SAMPLE	1	2	3	4	5
Del 3	-	-	+	+	-
igf	-	+	+	-	nrg

igf1r

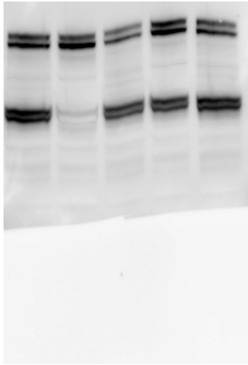

SAMPLE	1	2	3	4	5
Del 3	-	-	+	+	-
igf	-	+	+	-	nrg

B-actin

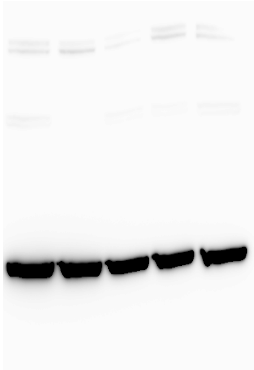

SAMPLE	1	2	3	4	5
Del 3	-	-	+	+	-
igf	-	+	+	-	nrg

erbB3

P-HER3/ErbB3

1 2 3 4

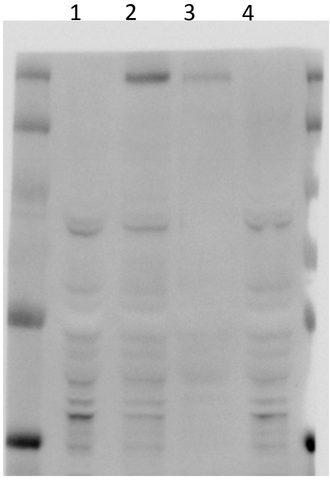

HER3/ErbB3

1 2 3 4

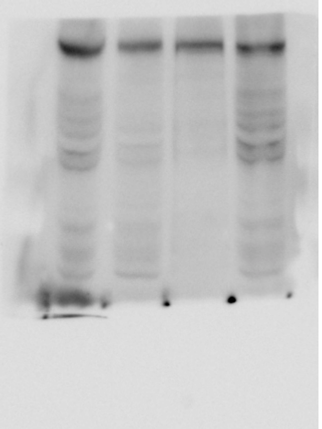

B-actin

1 2 3 4

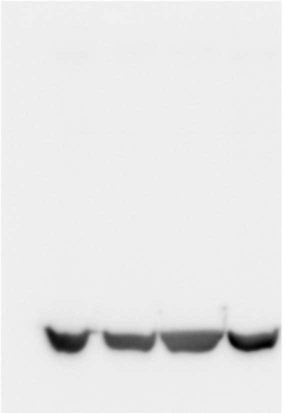

	1	2	3	4
Del 3	-	-	+	+
NRG	-	+	+	-

W4-165 repeat western

MET

P-met

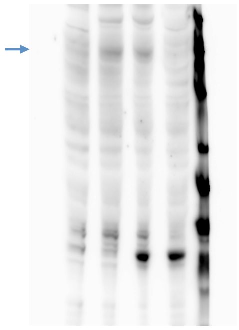

SAMPLE	1	2	3	4
Del 3	-	-	+	+
hgf	-	+	+	-

met

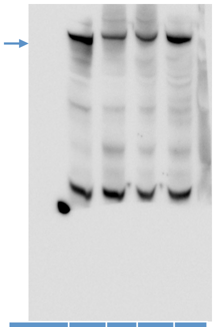

SAMPLE	1	2	3	4
Del 3	-	-	+	+
hgf	-	+	+	-

B-actin

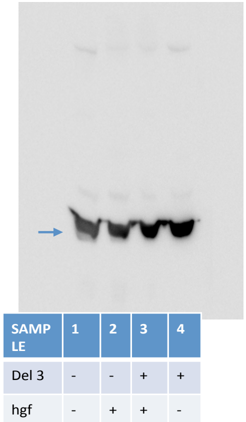

SAMPLE	1	2	3	4
Del 3	-	-	+	+
hgf	-	+	+	-

FGF receptor

P-FGFr

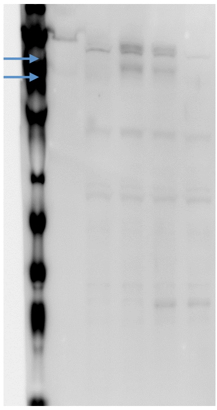

SAMPLE	1	5	6	7
Del 3	-	-	+	+
bFGF	-	+	+	-

FGFr

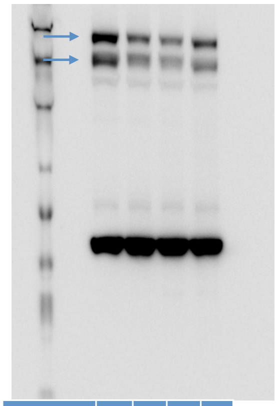

SAMPLE	1	5	6	7
Del 3	-	-	+	+
bFGF	-	+	+	-

B-actin

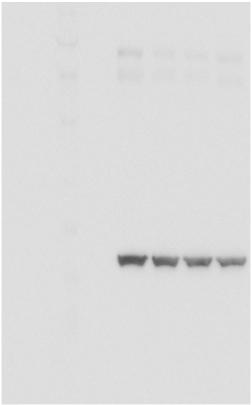

SAMPLE	1	5	6	7
Del 3	-	-	+	+
bFGF	-	+	+	-

PDGF receptor

P-PDGFr

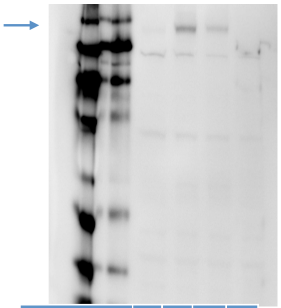

SAMPLE	1	2	3	4
Del 3	-	-	+	+
PDGF	-	+	+	-

PDGFr

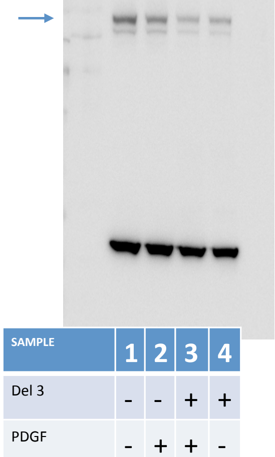

SAMPLE	1	2	3	4
Del 3	-	-	+	+
PDGF	-	+	+	-

B-actin

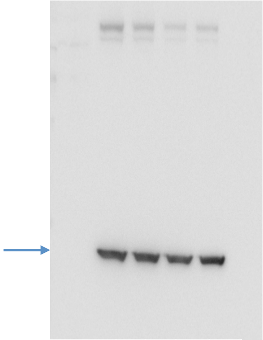

SAMPLE	1	2	3	4
Del 3	-	-	+	+
PDGF	-	+	+	-

Supplementary Figure S4.

EGFR

EGFR-P

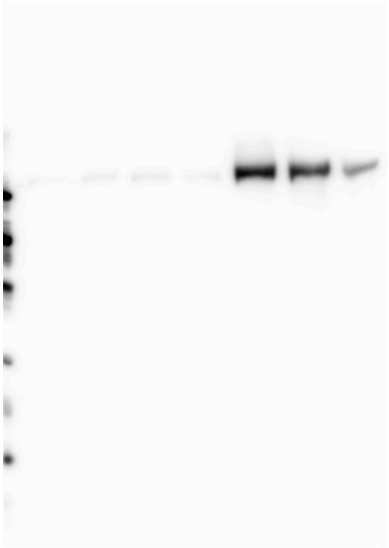

SAMPLE (5-83)	13	14	15	16	17	18	11
ERLOT	+	+	+	+	-	-	-
EGF	-	+	+	-	+	+	-
D3	-	-	+	+	-	+	-

EGFR

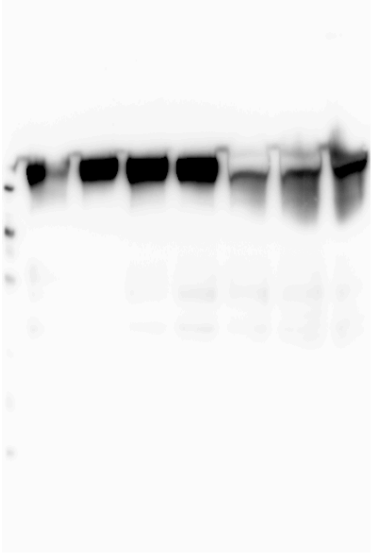

SAMPLE (5-83)	13	14	15	16	17	18	11
ERLOT	+	+	+	+	-	-	-
EGF	-	+	+	-	+	+	-
D3	-	-	+	+	-	+	-

ERK

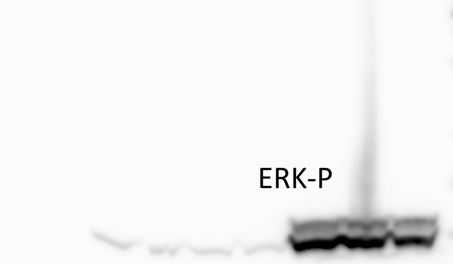

SAMPLE (5-83)	13	14	15	16	17	18	11
ERLOT	+	+	+	+	-	-	-
EGF	-	+	+	-	+	+	-
D3	-	-	+	+	-	+	-

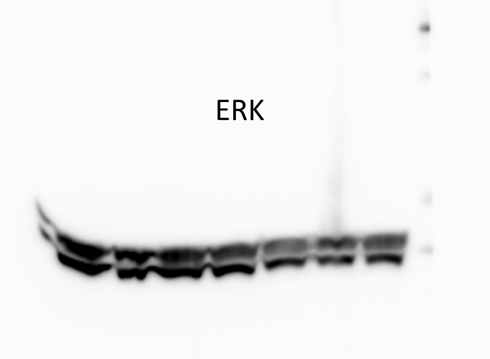

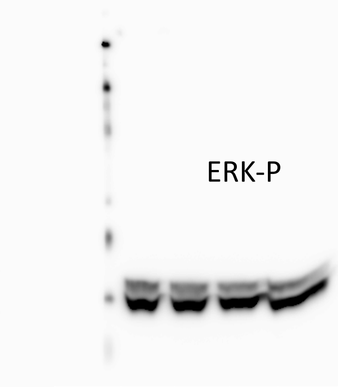

SAMPLE	1	4	5	6
Del 3	-	+	-	+
NRG	-	-	+	+

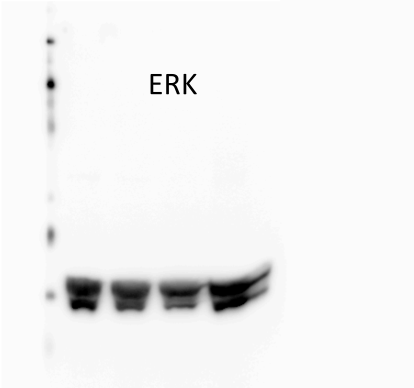

AKT

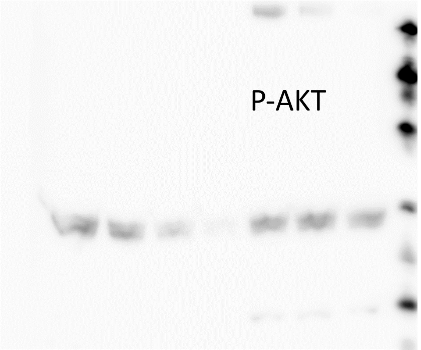

SAMPLE (5-83)	13	14	15	16	17	18	11
ERLOT	+	+	+	+	-	-	-
EGF	-	+	+	-	+	+	-
D3	-	-	+	+	-	+	-

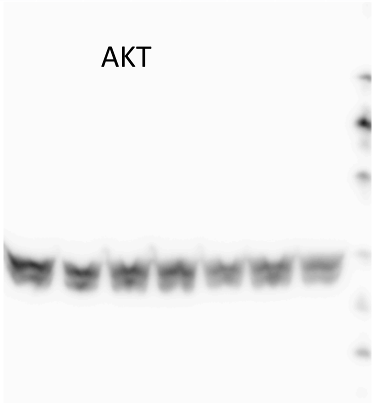

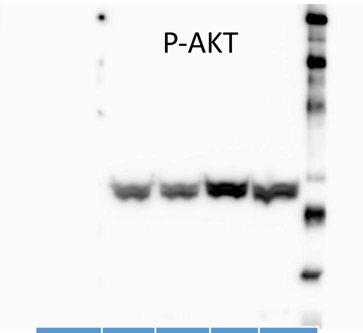

SAMPLE	1	4	5	6
Del 3	-	+	-	+
NRG	-	-	+	+

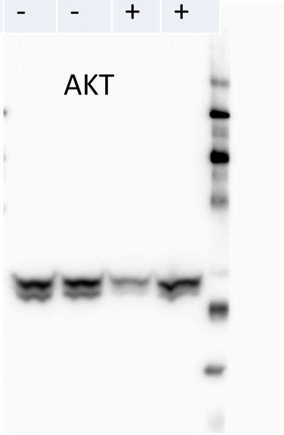

Supplement: Supplementary file 2 — Supplementary Information.2 [file 41598_2020_59466_MOESM2_ESM.pdf]
